# Effect of vitamin E intake on glycemic control and insulin resistance in diabetic patients: an updated systematic review and meta-analysis of randomized controlled trials

**DOI:** 10.1186/s12937-023-00840-1

**Published:** 2023-02-17

**Authors:** Omid Asbaghi, Behzad Nazarian, Mojtaba Yousefi, Javad Anjom-Shoae, Hamid Rasekhi, Omid Sadeghi

**Affiliations:** 1 Cancer Research Center, Student Research Committee, Shahid Beheshti University of Medical Sciences, Tehran, Iran; 2 Student Research Committee, Lorestan University of Medical Sciences, Khorramabad, Iran; 3 Department of Nutrition, School of Health and Nutrition, Yasuj University of Medical Sciences, Yasuj, Iran; 4 Adelaide Medical School and Centre of Research Excellence in Translating Nutritional Science to Good Health, University of Adelaide, Adelaide, SA 5005 Australia; 5 Department of Nutrition Research, National Nutrition and Food Technology Research Institute and Faculty of Nutrition Sciences and Food Technology, Shahid Beheshti University of Medical Sciences, Tehran, Iran; 6 Nutrition and Food Security Research Center and Department of Community Nutrition, School of Nutrition & Food Sciences, Isfahan University of Medical Sciences, Isfahan, Iran; 7 Student Research Committee, Isfahan University of Medical Sciences, Isfahan, Iran

**Keywords:** Diabetes mellitus; Blood glucose; HbA1c, Vitamin E, Tocopherol

## Abstract

Since a 2014 meta-analysis, several randomized controlled trials (RCTs) evaluating the effect of vitamin E intake on glycemic indices and insulin resistance in adults with diabetes have reached inconsistent conclusions. Therefore, we updated the previous meta-analysis to summarize the current evidence in this regard. Online databases including PubMed, Scopus, ISI Web of Science, and Google Scholar were searched to identify relevant studies published up to September 30, 2021, using relevant keywords. Random-effects models were used to obtain overall mean difference (MD) comparing vitamin E intake with a control group. In total, 38 RCTs with a total sample size of 2171 diabetic patients (1110 in vitamin E groups and 1061 in control groups) were included. Combining the results from 28 RCTs on fasting blood glucose, 32 RCTs on HbA1c, 13 RCTs on fasting insulin, and 9 studies on homeostatic model assessment for insulin resistance (HOMA-IR) showed a summary MD of -3.35 mg/dL (95% CI: -8.10 to 1.40, *P* = 0.16), -0.21% (95% CI: -0.33 to -0.09, *P* = 0.001), -1.05 µIU/mL (95% CI: -1.53 to -0.58, *P* < 0.001), and -0.44 (95% CI: -0.82 to -0.05, *P* = 0.02), respectively. This indicates a significant lowering effect of vitamin E on HbA1c, fasting insulin and HOMA-IR, while no significant effect on fasting blood glucose in diabetic patients. However, in subgroup analyses, we found that vitamin E intake significantly reduced fasting blood glucose in studies with an intervention duration of < 10 weeks. In conclusion, vitamin E intake has a beneficial role in improving HbA1c and insulin resistance in a population with diabetes. Moreover, short-term interventions with vitamin E have resulted in lower fasting blood glucose in these patients. This meta-analysis was registered in PROSPERO with code CRD42022343118.

**Supplementary Information:**

The online version contains supplementary material available at 10.1186/s12937-023-00840-1.

## Introduction

Diabetes mellitus, a chronic metabolic disorder, is associated with an increased risk of different comorbidities including cardiovascular disease (CVD), chronic kidney disease, retinopathy, and mortality as well [1]. Hyperglycemia, which is among the main signs of diabetes, has been shown to be involved in the development of vascular complications and subsequent disorders [2]. Prior studies have proved that controlling glycaemia is the best approach to prevent subsequent disorders among patients with diabetes [3]. Recently, it has been shown that supplementation with antioxidants, such as vitamin E, may ameliorate endothelial cell dysfunction in patients with diabetes [4, 5]. However, it is unclear whether this is mediated through the effect of vitamin E on glycemic indices and insulin resistance or other pathways. It should be noted that supplementation with these antioxidants has lower costs and is associated with fewer side effects compared with the regular drugs used to control diabetes.

There is evidence of a positive association between reduced levels of vitamin E and risk factors of type 2 diabetes mellitus (T2DM) including insulin resistance and hyperglycemia [6]. It has been proposed that vitamin E inhibits glucose oxidation which is a necessary step for protein glycosylation and producing hemoglobin A1c (HbA1c) [7]. Additionally, a prior meta-analysis of prospective cohort studies showed that a higher intake of foods rich in vitamin E (nuts, seeds, liquid oil, and raisin) was associated with a reduced risk of hyperglycemia and diabetes [8]. There is also further evidence indicating the beneficial effects of adherence to vitamin-E-rich diets on glycemic control in diabetic patients [9].

In contrast to that meta-analysis, findings from randomized controlled trials (RCTs) investigating the effect of vitamin E supplementation on glycemic control and insulin resistance in different types of diabetes are conflicting. Some studies showed that vitamin E supplementation improves glycemic indices and insulin resistance in patients with T2DM from Western and Asian countries [10–13], while other studies from these regions did not report such a significant effect on patients with diabetic nephropathy [14–16] and T2DM patients [17–19]. In contrast, some studies indicated a significant increase in the blood glucose level following vitamin E supplementation among Asian patients with T2DM [20, 21]. In a meta-analysis of RCTs in 2014, Xu et al. reported that vitamin E supplementation did not result in significant benefits in glycemic control, as measured by glycated hemoglobin (HbA1c), and fasting insulin, among patients with T2DM [22]. However, since the release of Xu et al. meta-analysis, 14 RCTs were published on the effect of vitamin E supplementation on glycemic indices in patients with different subtypes of diabetes [14, 17, 18, 21, 23–29]. Moreover, Xu et al. only included the RCTs conducted on T2DM patients and therefore, the effect of vitamin E on T1DM patients remained unclear. The current systematic review and meta-analysis of RCTs, therefore, was conducted to summarize available findings on the effect of vitamin E supplementation on glycemic indices and insulin resistance in patients with different subtypes of diabetes (T2DM, T1DM, diabetic nephropathy).

## Methods

This study was performed based on the PRISMA (Preferred Reporting Items for Systematic Reviews and Meta-Analyses) protocol for reporting systematic reviews and meta-analyses [30]. This study was registered in PROSPERO with code CRD42022343118.

### Search strategy

A comprehensive literature search was done using the online databases of PubMed, Scopus, and Web of Science until September 30, 2021, to identify RCTs that examined the effect of vitamin E supplementation on glycemic indices and insulin resistance in patients with either type 1 or type 2 diabetes mellitus. The keywords used in the search strategy are presented in Supplemental Table 1. We considered no language or time restriction in the systematic search. Reference lists of the selected articles were manually searched to avoid any missings of eligible publications. Also, a web-based search was conducted in Google Scholar using relevant terms. In this engine, we screened the first 500 relevancy-ranked papers.

### Inclusion criteria

We included studies that were randomized controlled clinical trials, performed on diabetic patients (T2DM, T1DM, diabetic nephropathy) with an age range of ≥ 18 years, prescribed vitamin E in different chemical forms including alpha-, beta-, gamma-, and delta-tocopherol and alpha-, beta-, gamma-, and delta-tocotrienol with any dosage and intensity (e.g. daily), had at least one week’s duration of intervention, and measured glycemic and insulin indices including fasting glucose (mg/dL), fasting insulin (µIU/mL), HbA1c (%), or HOMA-IR following vitamin E intake. Moreover, we only included studies that reported mean changes and their standard deviations (SDs) of glycemic indices for both intervention and control groups or those studies that presented required information (mean ± SD at the study baseline and end of the trial) for calculating these estimates as these effect sizes were required for performing the analyses. If data from one dataset were published in > 1 article, the one with more complete findings or a greater number of participants was included.

### Exclusion criteria

In the current meta-analysis, we did not include open clinical trials and semi-experimental studies, those with a cohort, cross-sectional, and case–control design, review articles, and ecological studies. Clinical trials without a placebo or control group and those that were performed on children or adolescents were excluded as well. We also excluded studies that administered vitamin E in combination with other nutrients, as it was impossible to consider the effect of vitamin E alone. RCTs, performed on women with gestational diabetes mellitus, were also excluded.

### Study selection and data extraction

First, all articles, found through the online databases, were included in Endnote X9, and then, by considering the title and abstract of articles, two independent reviewers (OA and BN) screened the articles based on the inclusion and exclusion criteria. For possible eligible articles, their full texts were also reviewed. Finally, the articles, chosen by the two reviewers, were assessed. Any disagreement between the two reviewers was resolved with a third reviewer (OS). An Excel-based form was designed to extract data from each article. This form was used in our previous meta-analyses [3, 31, 32] and was revised based on the current title. We extracted the following data: name of the first author, publication year, demographic characteristics (mean age and sex distribution), design, sample size (control and intervention groups), type of vitamin E prescribed, vitamin E dosage, duration of the intervention, mean changes and their SDs of outcomes (glycemic and insulin indices) for the intervention and control groups, and the confounding variables adjusted in the analyses. Data extraction was done by two independent investigators (BN and MY) and any disagreement was resolved by discussion. A third reviewer (OS) double-checked the extracted data and finalized them for statistical analysis. If an article had missing data or its full text was not available, we contacted its authors to obtain the required data.

### Primary and secondary outcome variables

In the present meta-analysis, fasting glucose, HbA1c, and fasting insulin were considered as primary outcomes and HOMA-IR was a secondary outcome. HOMA-IR was used as an index to quantify insulin resistance and beta-cell function considering the fasting levels of both insulin and glucose [33]. Mean changes and their SDs of outcomes during the intervention period in both vitamin E and control groups were included in the statistical analysis. If the data of each outcome was reported in different units, we converted that to the most frequently used unit (mg/dL for fasting glucose, percent for HbA1c, µIU/mL for fasting insulin).

### Risk of bias assessment

Cochrane quality assessment tool was used to assess the risk of bias for each study [34]. This tool contained seven domains including random sequence generation, allocation concealment, reporting bias, performance bias, detection bias, attrition bias, and other sources of bias. Each domain was given a “high risk” score if the study comprised methodological defects which may have affected its findings, a “low risk” score if there was no defect for that domain, and an “unclear risk” score if the information was not sufficient to determine the impact. If the trial had “low risk” for all domains, it was considered a high-quality study with a totally low risk of bias. A risk of bias assessment was done by two independent investigators (OA and BN).

### Statistical analysis

Mean changes and their SDs of glycemic measures following vitamin E supplementation, compared with a control group, were included in the meta-analysis. When mean changes were not available, we calculated them by considering changes in glycemic measures during the intervention. We also converted standard errors (SEs), 95% confidence intervals (CIs), and interquartile ranges (IQRs) to SDs using the method of Hozo et al. [35]*.* To obtain the overall mean difference (MD), we applied a random-effects model that takes both within and between-study variations into account. I^2^ statistic and Cochrane’s Q test were used to assess heterogeneity among studies. For the I^2^ statistic, we considered I^2^ values of < 25%, 25–50%, 50–75%, and > 75% as low, moderate, high, and very high between-study heterogeneity, respectively [36, 37]. To find probable sources of heterogeneity, subgroup analyses were performed. Subgroup analyses were conducted based on study locations (Western vs. non-Western countries), study designs (blinded vs. not-blinded RCTs and cross-over vs. parallel RCTs), duration of the intervention (≥ 10 vs. < 10 weeks), types (alpha-tocopherol vs. other forms) and dosages of vitamin E (≥ 500 vs. < 500 mg/day), types of diabetes (type 1 vs. type 2 diabetes vs. diabetic nephropathy), and risk of bias (high vs. low). To determine the non-linear effects of vitamin E dosage (mg/d) on glycemic measures, fractional polynomial modeling was applied. To detect the dependency of the overall effect size on a particular study, we performed a sensitivity analysis using the “metaninf” command, in which the overall effect size was obtained after excluding each study. This was a priori sensitivity analysis. Also, to obtain the overall effect sizes without considering the RCTs with combination treatment (i.e. vitamin E + basic treatment versus basic treatment), we conducted an additional analysis by excluding those RCTs. The possibility of publication bias was examined using the Egger regression test. In case of any significant publication bias, the trim-and-fill method was conducted. The meta-analysis was carried out using Stata, version 11.2 (StataCorp). *P* value < 0.05 was considered as significant.

## Results

Findings from the literature search: In our systematic search, 2340 papers in different databases were found, of them, 760 were duplicates and 1536 were unrelated by screening their titles and abstracts. In total, 44 articles remained for the full-text assessment. Among them, three studies were excluded because they were conducted on children [38–40]. The study of Farvid et al. was also excluded because they examined the combined effects of vitamins E and C, not the effect of vitamin E alone, on diabetic patients [41]. One quasi-experimental study which had no control group was also excluded [42]. We excluded the study of Rajanandh et al. in which patients in the control group received pregabalin with oral hypoglycemic agents rather than a placebo [43]. Finally, 38 RCTs containing complete data on the effect of vitamin E intake on glycemic or insulin indices were included in this systematic review and meta-analysis [7, 10–21, 23–29, 44–61]. The flow diagram of the study selection is provided in Supplemental Fig. 1. Among these studies, 28 studies assessed the effect of vitamin E on fasting blood glucose [7, 10–13, 17–21, 23, 25–28, 44, 45, 47–49, 51, 53–57, 60, 61], 32 studies on HbA1c [7, 10, 12–16, 18, 20, 21, 23–26, 28, 29, 44, 46, 48–61], 13 studies on fasting insulin [12, 17–19, 21, 23, 26–28, 45, 47, 49, 51, 60], and 9 studies on HOMA-IR [12, 17, 18, 21, 26–28, 45, 51].

### Characteristics of included studies

We described the characteristics of RCTs included in the current meta-analysis in Table 1. These studies were published between 1988 and 2021 and included a total sample size of 2171 patients with diabetes (1110 in the vitamin E group and 1061 in the control group). Most studies recruited male and female patients; however, 3 RCTs were done on males [26, 47, 57] and one on females only [20]. Of the 38 included studies, 16 studies were conducted in Western countries [7, 10, 18–20, 23, 46, 47, 51–57, 59] including the US (*n* = 4), Europe (*n* = 10), and Australia (*n* = 2), and the remaining studies were performed in Asia [11–17, 21, 24–29, 44, 48–50, 58, 60, 61] and Brazil [45]. Six studies had a crossover design [10, 46, 48, 52, 53, 59] and others were parallel. In terms of types of diabetes, 7 studies included patients with type 1 diabetes [7, 46, 52, 53, 55, 56, 59], 3 studies recruited patients with diabetic nephropathy [14, 17, 29], one study perform the intervention on patients with diabetic neuropathy [15], and 26 studies enrolled type 2 diabetic patients. One study recruited patients with type 1 or 2 diabetes [50]. Patients in 30 RCTs were blinded to the interventions, while in 8 studies, participants were aware of the intervention type [7, 20, 25, 47, 55, 56, 58, 60]. Regarding the vitamin E types, 19 studies used alpha-tocopherol for the intervention, one study did intervention using mixed alpha- and gamma-tocopherols [19], and 6 studies prescribed tocotrienols for patients [14–16, 18, 29, 48]. In the remaining 12 studies, the type of vitamin E administered was unclear [7, 12, 13, 17, 23, 25, 27, 52, 53, 58, 59, 61]. Vitamin E dosages in included studies varied from 90 to 1620 mg/day. Among the 28 articles, three evaluated dietary intake of vitamin E throughout the trial and reported a non-significant difference between vitamin E and placebo groups in this regard [12, 21, 60]. The duration of intervention was between 4 and 52 weeks among the included RCTs. Regarding compliance, one studies assessed serum levels of vitamin E before and after the intervention [47], 12 studies evaluated compliance by considering prescribed and consumed vitamin E supplements throughout the trial (consumed supplements /prescribed supplements *100) [10, 12–14, 16, 20, 23–25, 28, 29, 50], two studies evaluated compliance by phone call [20, 60], and the remaining studies did not assess compliance [17–19, 46, 47, 53–55]. High adherence to vitamin E intake was reported in all studies that assessed compliance, while it was not clear among other studies not considering compliance. Of 34 studies, 8 reported adjusted effect sizes for the effect of vitamin E on the outcomes [11, 12, 16, 17, 19, 21, 24, 28], while the remaining RCTs presented non-adjusted estimates. Regarding the risk of bias assessment, five studies had a low risk of bias [12, 18, 21, 23, 28], whereas others had a high or unclear risk of bias in at least one aspect of the Cochrane risk of bias tool (Supplemental Table 2).

**Table 1:** Summary of randomized controlled trials on the effects of vitamin E supplementation on glycemic indices and insulin resistance in patients with diabetes mellitus

Author, year	Design	Participants, n	Diabetes Type	Age, year^a^	Intervention		Duration (week)	Outcomes (changes)^b^		Adjust/matching^3^
					Treatment group	Control group		Treatment group	Control group	
Koay et al. 2021 [14]	RA/PC/DB / PA	M/F: 59: Int: 31, Con: 28	Diabetic nephropathy	Int: 66 ± 13, Con:70 ± 13	400 mg/d tocotrienol-Rich Vitamin E	Placebo: NR	52	HbA1c: 0.16 ± 0.86%	HbA1c: 0.42 ± 0.74%	Gender, duration of diabetes, baseline HbA1c
Tat-Ng et al. 2020	RA/PC/DB / PA	M/F: 80: Int: 39, Con: 41	Diabetic neuropathy	Int: 63 ± 12, Con:64 ± 15	400 mg/d tocotrienol-Rich Vitamin E	Placebo: NR	8	HbA1c: 0.23 ± 1.05%	HbA1c: 0.41 ± 1.12%	
Dalan et al. 2020 [24]	RA/PC/DB / PA	M/F: 166: Int: 84, Con: 82	T2DM	Int: 55 ± 10, Con: 57 ± 10	266 mg/d α-tocopherol	Placebo: NR	24	HbA1c: -0.08 ± 0.73%	HbA1c: -0.03 ± 0.82%	Baseline alpha-tocopherol, haptoglobin genotype
Jie-Tan et al. 2019	RA/PC/DB / PA	M/F: 54: Int: 27, Con: 27	Diabetic nephropathy	Int: 59 ± 10, Con:62 ± 11	400 mg/d tocotrienol-Rich Vitamin E	Placebo: NR	12	HbA1c: -0.6 ± 0.95%	HbA1c: -0.38 ± 0.89%	Gender, duration of diabetes, baseline HbA1c
Bril et al. 2019 [23]	RA/PC/DB / PA	M/F: 68: Int: 36, Con: 32	T2DM	Int: 60 ± 9, Con:57 ± 11	720 mg/d vitamin E	Placebo: NR	77	Insulin: -3.0 ± 6.0 µIU/mL FBG: -3.0 ± 39.0 mg/dL HbA1c: -0.3 ± 1.2%	Insulin: 3.0 ± 12.0 µIU/mL FBG: 6.0 ± 53.0 mg/dL HbA1c: 0.3 ± 1.6%	
El-Aal et al. 2018 [26]	RA/PC/SB/ PA	M: 20: Int: 10, Con: 10	T2DM	Int: 51, Con: 51	400 mg/d α-tocopherol + metformin	Placebo: metformin	12	Insulin: -3.8 ± 3.4 µIU/mL FBG: -23.1 ± 13.6 mg/dL HbA1c: -0.9 ± 1.2% HOMA: -4.7 ± 1.1	Insulin: 1.7 ± 5.0 µIU/mL FBG: 14.2 ± 23.6 mg/dL HbA1c: 0.06 ± 0.8% HOMA: 1.5 ± 2.7	
		M: 20: Int: 10, Con: 10	T2DM	Int: 51, Con: 51	400 mg/d α-tocopherol + metformin + vitamin C	Placebo: metformin + vitamin C	12	Insulin: -5.0 ± 5.3 µIU/mL FBG: -29.2 ± 33.0 mg/dL HbA1c: -1.0 ± 0.8% HOMA: -4.5 ± 1.2	Insulin: -4.3 ± 2.9 µIU/mL FBG: -24.5 ± 23.1 mg/dL HbA1c: -0.7 ± 0.6% HOMA: -2.3 ± 1.2	
Tan et al. 2018	RA/PC/DB / PA	M/F: 45: Int: 22, Con: 23	T2DM	Int: 59 ± 10, Con:63 ± 10	400 mg/d tocotrienol-rich vitamin E	Placebo: NR	8	HbA1c: -0.55 ± 1.8%	HbA1c: -0.09 ± 1.3%	Age, baseline values
Dass et al. 2018 [25]	RA/PA	M/F: 60: Int: 31, Con: 29	T2DM	Int: 51 ± 9, Con:51 ± 7	400 mg/d vitamin E	Placebo: NR	12	FBG: -16.8 ± 18.3 mg/dL HbA1c: -0.19 ± 0.40%	FBG: -11.5 ± .19.2 mg/dL HbA1c: -0.31 ± 0.49%	
Rafraf et al. 2016 [28]	RA/PC/DB / PA	M/F: 83: Int: 42, Con: 41	T2DM	Int: 53 ± 6, Con:53 ± 8	360 mg/d α-tocopherol	Placebo: NR	8	Insulin: -1.3 ± 2.4 µIU/mL FBG: -9.1 ± 20.8 mg/dL HbA1c: -0.31 ± 0.67% HOMA: -0.66 ± 0.85	Insulin: -0.91 ± 2.90 µIU/mL FBG: 4.5 ± 19.1 mg/dL HbA1c: -0.19 ± 0.77% HOMA: -0.19 ± 1.13	Gender, Age, BMI, medication,FBS level
Stonehouse et al. 2016 [18]	RA/PC/DB / PA	M/F: 57: Int: 28, Con: 29	T2DM	Int: 60 ± 6, Con:61 ± 5	1020 mg/d α-tocopherol	Placebo: Palm olein	8	Insulin: -1.6 ± 0.7 µIU/mL FBG: -5.4 ± 13.8 mg/dL HbA1c: 0.02 ± 0.35% HOMA: -0.21 ± 0.57	Insulin: -0.78 ± 0.99 µIU/mL FBG: -1.9 ± 13.6 mg/dL HbA1c: 0.04 ± 0.34% HOMA: -0.11 ± 0.54	
Keihan et al. 2016 [44]	RA/PC/DB / PA	M/F: 68: Int: 32, Con: 36	DM	Int: 57 ± 6, Con:61 ± 6	150 mg/d α-tocopherol	Placebo: NR	12	FBG: -7.1 ± 12.3 mg/dL HbA1c: 0.54 ± 0.25%	FBG: -21.0 ± .7.1 mg/dL HbA1c: 0.4 ± 0.18%	
Khatami et al. 2016 [17]	RA/PC/DB / PA	M/F: 60: Int: 30, Con: 30	Diabetic nephropathy	Int: 61 ± 10, Con:62 ± 13	804 mg/d vitamin E	Placebo: NR	12	Insulin: -0.50 ± 2.19 µIU/mL FBG: 11.7 ± 31.2 mg/dL HOMA: 0.10 ± 1.64	Insulin: 0.70 ± 2.19 µIU/mL FBG: 1.7 ± 31.2 mg/dL HOMA: 0.30 ± 1.64	Gender, Age, medications, duration of diabetes, baseline BMI
Hejazi et al. 2015 [27]	RA/PC/SB / PA	M/F: 27: Int: 14, Con: 13	T2DM	Int: 48 ± 6, Con:46 ± 7	360 mg/d vitamin E	Placebo: NR	6	Insulin: 0.2 ± 3.3 µIU/mL FBG: -8.5 ± 42.5 mg/dL HOMA: 0.01 ± 1.22	Insulin: 2.7 ± 4.6 µIU/mL FBG: -16.0 ± 26.7 mg/dL HOMA: 0.06 ± 1.69	
Hashemi et al. 2014 [21]	RA/PC/DB / PA	M/F: 66: Int: 32, Con: 36	T2DM	Int: 44 ± 4, Con:45 ± 4	360 mg/d α-tocopherol acetate	Placebo: NR	12	Insulin: 0.3 ± 2.5 µIU/mL FBG: 36.1 ± 39.6 mg/dL HbA1c: 0.20 ± 0.85% HOMA: 0.50 ± 1.02	Insulin: 1.5 ± 2.8 µIU/mL FBG: 4.7 ± 35.1 mg/dL HbA1c: 0.2 ± 1.11% HOMA: 0.70 ± 1.15	
		M/F: 68: Int: 34, Con: 34	T2DM	Int: 45 ± 4, Con:44 ± 5	360 mg/d α-tocopherol acetate + EPA	Placebo + EPA	12	Insulin: -1.6 ± 2.4 µIU/mL FBG: -10.1 ± 31.7 mg/dL HbA1c: -0.6 ± 0.85% HOMA: -0.7 ± 1.08	Insulin: -0.6 ± 3.6 µIU/mL FBG: -19.5 ± 23.2 mg/dL HbA1c: -0.8 ± 0.79% HOMA: -0.6 ± 1.26	
Shadman et al. 2013 [12]	RA/PC/DB / PA	M/F: 46: Int: 17, Con: 29	Overweight T2DM	Int: 47 ± 4, Con:45 ± 6	90 mg/d vitamin E + CLA	Placebo: CLA	8	Insulin: -0.30 ± 4.15 µIU/mL FBG: -4.2 ± 10.7 mg/dL HbA1c: -0.50 ± 1.00% HOMA: -0.27 ± 1.94	Insulin: 0.50 ± 2.09 µIU/mL FBG: 2.7 ± 12.5 mg/dL HbA1c: -1.01 ± 1.04% HOMA: -0.7 ± 1.70	Body composition
Rafraf et al. 2012 [11]	RA/PC/DB / PA	M/F: 83: Int: 42, Con: 41	T2DM	Int: 35 ± 6, Con:35 ± 8	400 mg/d α-tocopherol acetate	Placebo: NR	8	FBG: -9.1 ± 21.3 mg/dL	FBG: 4.5 ± 19.4 mg/dL	Gender, Age, BMI, duration of diabetes, baseline values
Udupa et al. 2012 [13]	RA/PC/DB / PA	M/F: 50: Int: 25, Con: 25	T2DM	Int: 53 ± 2, Con:53 ± 2	400 mg/d vitamin E	Placebo: NR	12	FBG: -6.2 ± 14.4 mg/dL HbA1c: -1.54 ± 1.14%	FBG: 4.9 ± 14.6 mg/dL HbA1c: -0.41 ± 1.16%	
Vijayakumar et al. 2011	RA/ PA	M/F: 74 Int: 38, Con: 36	T2DM	Int: 54 ± 8, Con:56 ± 8	600 mg/d vitamin E	Placebo: NR	13	HbA1c: -1.16 ± 0.77%	HbA1c: -0.99 ± 0.77%	
Oliveira et al. 2011 [45]	RA/PC/DB / PA	M/F: 51 Int: 25, Con: 26	T2DM	30–79	800 mg/d α-tocopherol	Placebo: NR	17	Insulin: 0.80 ± 5.69 µIU/mL FBG: -1.0 ± 34.94 mg/dL HOMA: 0.40 ± 1.98	Insulin: 0.00 ± 4.84 µIU/mL FBG: -0.30 ± 46.49 mg/dL HOMA: -0.20 ± 2.32	
		M/F: 51 Int: 25, Con: 26	T2DM	30–79	800 mg/d α-tocopherol + lipoic acid	Placebo + lipoic acid	17	Insulin: 2.1 ± 4.7 µIU/mL FBG: -17.7 ± 42.5 mg/dL HOMA: 0.10 ± 1.26	Insulin: -0.9 ± 6.69 µIU/mL FBG: -13.2 ± 57.9 mg/dL HOMA: -0.6 ± 3.51	
Giannini et al. 2007 [46]	RA/PC/DB/ CO	M/F: 10 Int: 10, Con: 10	T1DM	Int: 18 ± 3, Con: 18 ± 3	1200 mg/d α-tocopherol	Placebo: NR	24	HbA1c: -0.04 ± 0.37%	HbA1c: -0.02 ± 0.78%	
Winterbone et al. 2007 [47]	RA/PC/ PA	M/F: 19 Int: 10, Con: 9	T2DM	Int: 62 ± 5, Con: 61 ± 5	1080 mg/d α-tocopherol	Placebo: NR	4	Insulin: 6.6 ± 19.2 µIU/mL FBG: -9.0 ± 32.6 mg/dL	Insulin: -1.0 ± 14.1 µIU/mL FBG: 1.8 ± 13.5 mg/dL	
Ward et al. 2007 [19]	RA/PC/DB / PA	M/F: 36 Int: 18, Con: 18	T2DM	Int: 64 ± 29, Con: 62 ± 29	500 mg/d α-tocopherol	Placebo: NR	6	Insulin: 1.1 ± 3.3 µIU/mL FBG: -3.6 ± 26.0 mg/dL	Insulin: -1.3 ± 6.2 µIU/mL FBG: -1.7 ± 28.1 mg/dL	Blood pressure
		M/F: 37 Int: 19, Con: 18		Int: 58 ± 17, Con: 62 ± 29	500 mg/d mixed tocopherols	Placebo: NR		Insulin: -0.10 ± 4.56 µIU/mL FBG: 7.2 ± 33.2 mg/dL	Insulin: -1.30 ± 6.20 µIU/mL FBG: -1.7 ± 28.1 mg/dL	
Baliarsingh et al. 2005 [48]	RA/PC/DB/ CO	M/F: 19 Int: 19, Con: 19	T2DM	Int: 48 ± 6, Con: 52 ± 6	180 mg/d α-tocopherol	Placebo: NR	8	FBG: 1.4 ± 10.1 mg/dL HbA1c: 0.00 ± 0.25%	FBG: 5.9 ± 13.0 mg/dL HbA1c: 0.00 ± 0.25%	
Boshtam et al. 2005 [49]	RA/PC/TB / PA	M/F: 100 Int: 50, Con: 50	T2DM	Int: 52 ± 9, Con: 54 ± 7	134 mg/d α-tocopherol	Placebo: NR	27	Insulin: 2.5 ± 6.9 µIU/mL FBG: -8.5 ± 36.9 mg/dL HbA1c: -0.40 ± 0.96%	Insulin: 2.1 ± 5.5 µIU/mL FBG: -27.0 ± 59.3 mg/dL HbA1c: 0.10 ± 1.01%	Gender, age, education, occupation
Ble-Castillo et al. 2005 [20]	RA/PC/PA	F: 34:: Int: 13, Con: 21	T2DM	Int: 51 ± 14, Con: 55 ± 11	800 mg/d α-tocopherol	Placebo: NR	6	FBG: 17.1 ± 14.5 mg/dL	FBG: -32.9 ± 53.8 mg/dL	
Economides et al. 2005 [50]	RA/PC/ DB/ PA	M/F: 66: Int: 34, Con: 32	T2DM	Int: 53 ± 14, Con: 53 ± 14	1080 mg/d vitamin E	Placebo: NR	52	HbA1c: 0.00 ± 0.74%	HbA1c: 0.00 ± 0.66%	
Manzella et al. 2001 [51]	RA/PC/DB / PA	M/F: 50: Int: 25, Con: 25	T2DM	Int: 63 ± 5, Con: 65 ± 4	600 mg/d α-tocopherol	Placebo: NR	17	Insulin: -11.1 ± 0.1 µIU/mL FBG: -1.8 ± 3.1 mg/dL HbA1c: -0.70 ± 0.18% HOMA: -0.53 ± 0.23	Insulin: -0.4 ± 0.1 µIU/mL FBG: -1.8 ± 2.4 mg/dL HbA1c: -0.10 ± 0.36% HOMA: -0.01 ± 0.13	
Park et al. 2001	RA/PC/PA	M/F: 98 Int: 58, Con: 40	T2DM	Int: 49 ± 9, Con: 49 ± 10	200 mg/d α-tocopherol + CSII	Placebo: CSII	8	Insulin: 6.1 ± 3.5 µIU/mL FBG: -91.8 ± 80.1 mg/dL HbA1c: -3.5 ± 1.3%	Insulin: 7.9 ± 1.3 µIU/mL FBG: -88.2 ± .65.3 mg/dL HbA1c: -3.3 ± 1.3%	
Feng et al. 2000 [52]	RA/PC/DB/ CO	M/F: 20 Int: 20, Con: 20	T2DM	Int: 32 ± 8, Con: 32 ± 8	1620 mg/d vitamin E	Placebo: NR	17	HbA1c: 0.33 ± 0.80%	HbA1c: 0.18 ± 0.87%	
Bursell et al. 1999 [53]	RA/PC/DB/ CO	M/F: 36 Int: 36, Con: 36	DM1	Int: 31 ± 7, Con: 31 ± 7	1206 mg/d vitamin E	Placebo: NR	36	FBG: -17.2 ± 65.1 mg/dL HbA1c: 0.20 ± 1.02%	FBG: -31.7 ± 62.3 mg/dL HbA1c: 0.20 ± 1.27%	
Gazis et al. 1999 [54]	RA/PC/DB / PA	M/F: 48 Int: 23, Con: 25	T2DM	Int: 56 ± 11, Con: 57 ± 11	1440 mg/d α-tocopherol	Placebo: NR	8	FBG: 2.3 ± 33.7 mg/dL HbA1c: 0.20 ± 0.98%	FBG: 5.4 ± 36.7 mg/dL HbA1c: 0.00 ± 0.67%	
Tutuncu et al. 1998	RA/PC/DB / PA	M/F: 48 Int: 23, Con: 25	T2DM	Int: 56 ± 11, Con: 57 ± 11	900 mg/d vitamin E	Placebo: NR	24	FBG: 9.0 ± 24.1 mg/dL HbA1c: -1.80 ± 1.07%	FBG: 7.2 ± 11.7 mg/dL HbA1c: 1.10 ± 2.02%	Age, duration of disease, metabolic control
Colette et al. 1998	RA/PC/DB/ CO	M/F: 9 Int: 9, Con: 9	T1DM	Int: 51 ± 11, Con: 51 ± 11	1000 mg/d vitamin E	Placebo: NR	5	HbA1c: 0.00 ± 0.94%	HbA1c: 0.70 ± 1.36%	Age, gender, weight
Duntas et al. 1996 [55]	RA/PC/PA	M/F: 24: Int: 12, Con: 12	T1DM	Int: 41 ± 12, Con:39 ± 12	360 mg/d α-tocopherol	Placebo: NR	26	FBG: -1.8 ± 19.7 mg/dL HbA1c: -0.12 ± 0.51%	FBG: 0.0 ± 19.7 mg/dL HbA1c: 0.7 ± 0.80%	Duration of disease, weight, glycemic control, insulin dose
		M/F: 24: Int: 12, Con: 12			720 mg/d α-tocopherol	Placebo: NR		FBG: -3.6 ± 18.7 mg/dL HbA1c: -0.56 ± 0.46%	FBG: 0.0 ± 19.7 mg/dL HbA1c: 0.7 ± 0.80%	
Fuller et al. 1996 [56]	RA/PC/PA	M/F: 30 Int: 15, Con: 15	T1DM	Int: 47 ± 14, Con: 47 ± 12	1080 mg/d α-tocopherol	Placebo: NR	8	FBG: 27.0 ± 68.0 mg/dL HbA1c: -0.30 ± 1.14%	FBG: 27.0 ± 46.5 mg/dL HbA1c: 0.40 ± 1.59%	
Reaven et al. 1995 [57]	RA/PC/DB / PA	M: 21: Int: 10, Con: 11	T2DM	Int: 60 ± 6, Con:61 ± 8	1440 mg/d α-tocopherol	Placebo: NR	10	FBG: -0.72 ± 22.76 mg/dL HbA1c: 0.20 ± 0.67%	FBG: -2.1 ± 21.3 mg/dL HbA1c: 0.00 ± 0.66%	
Paolisso et al. 1993 [10]	RA/PC/DB/ CO	NR: 25: Int: 25, Con: 25	T2DM	Int: 71, Con:71	900 mg/d d-a-tocopherol Ephynal	Placebo: NR	12	FBG: -12.6 ± 24.1 mg/dL HbA1c: -0.60 ± 1.58%	FBG: 1.8 ± 21.6 mg/dL HbA1c: 0.10 ± 1.58%	
Ceriello et al. 1991 [7]	RA/PC/PA	M/F: 20: Int: 10, Con: 10	T2DM	Int: 41 ± 4, Con:40 ± 4	1200 mg/d vitamin E	Placebo: NR	8	FBG: 9.0 ± 43.1 mg/dL HbA1c: -4.3 ± 1.3%	FBG: -1.8 ± 41.9 mg/dL HbA1c: -0.10 ± 1.39%	Age, duration of disease, metabolic control
			T2DM	Int: 42 ± 3, Con:40 ± 4	600 mg/d vitamin E	Placebo: NR		FBG: 10.8 ± 45.3 mg/dL HbA1c: -2.9 ± 1.9%	FBG: -1.8 ± 41.99 mg/dL HbA1c: -0.10 ± 1.39%	

Abbreviations: *FBG* Fasting blood glucose, *HbA1C* Hemoglobin A1c, *Int* Intervention, *Con* Control, *PA* Parallel, *CO* Crossover, *PC* Placebo Control, *RA* Randomized, *DB* Double blind, *TB* Triple blind, *M* Male, *F* Female, *DM* diabetes mellitus, *T2DM* Type 2 diabetes mellitus, *T1DM* Type 1 diabetes mellitus, *HOMA-IR* homeostatic model assessment for insulin resistance

^a^Values are mean ± SD or range (for age)

^b^Changes in concentrations of Insulin, HbA1C, Glucose, HOMA

### Findings from the systematic review

Out of the 28 articles that assessed the effect of vitamin E on fasting blood glucose, seven studies reported a significant lowering effect [10–13, 26, 28, 44], two studies showed a significant increasing effect [20, 21], and other studies did not show any significant effects. For HbA1c, eight studies indicated that vitamin E intake resulted in a significant reduction in HbA1c [7, 13, 26, 48, 49, 51, 61], one showed a significant increase in HbA1c [44], and other trials reported no significant effects. Five RCTs revealed that vitamin E intake significantly reduced fasting insulin [17, 18, 23, 26, 51], while other studies showed a non-significant effect. In terms of HOMA-IR, only three studies showed a significant reducing effect of vitamin E [11, 26, 51] and other trials failed to find a significant effect.

### Findings from the meta-analysis

All RCTs assessed in the systematic review were included in the meta-analysis. Of 38 RCTs, four had a combination treatment meaning that in the intervention group, vitamin E was prescribed with a basic treatment such as metformin, eicosapentaenoic acid, or lipoic acid, and in the control group, the basic treatment was prescribed only [12, 21, 26, 45]. On the other hand, the only difference between the two groups was vitamin E intake. Since the interaction between vitamin E and basic treatments may distort our findings, we conducted the analyses with and without the RCTs with a combination treatment. Also, three RCTs had two vitamin E groups with different types or dosages of vitamin E and one control group [7, 19, 55]. To avoid double-counting data, we assigned half of the controls to each vitamin E group in the meta-analysis.

### Vitamin E and fasting blood glucose

**Fig. 1:**
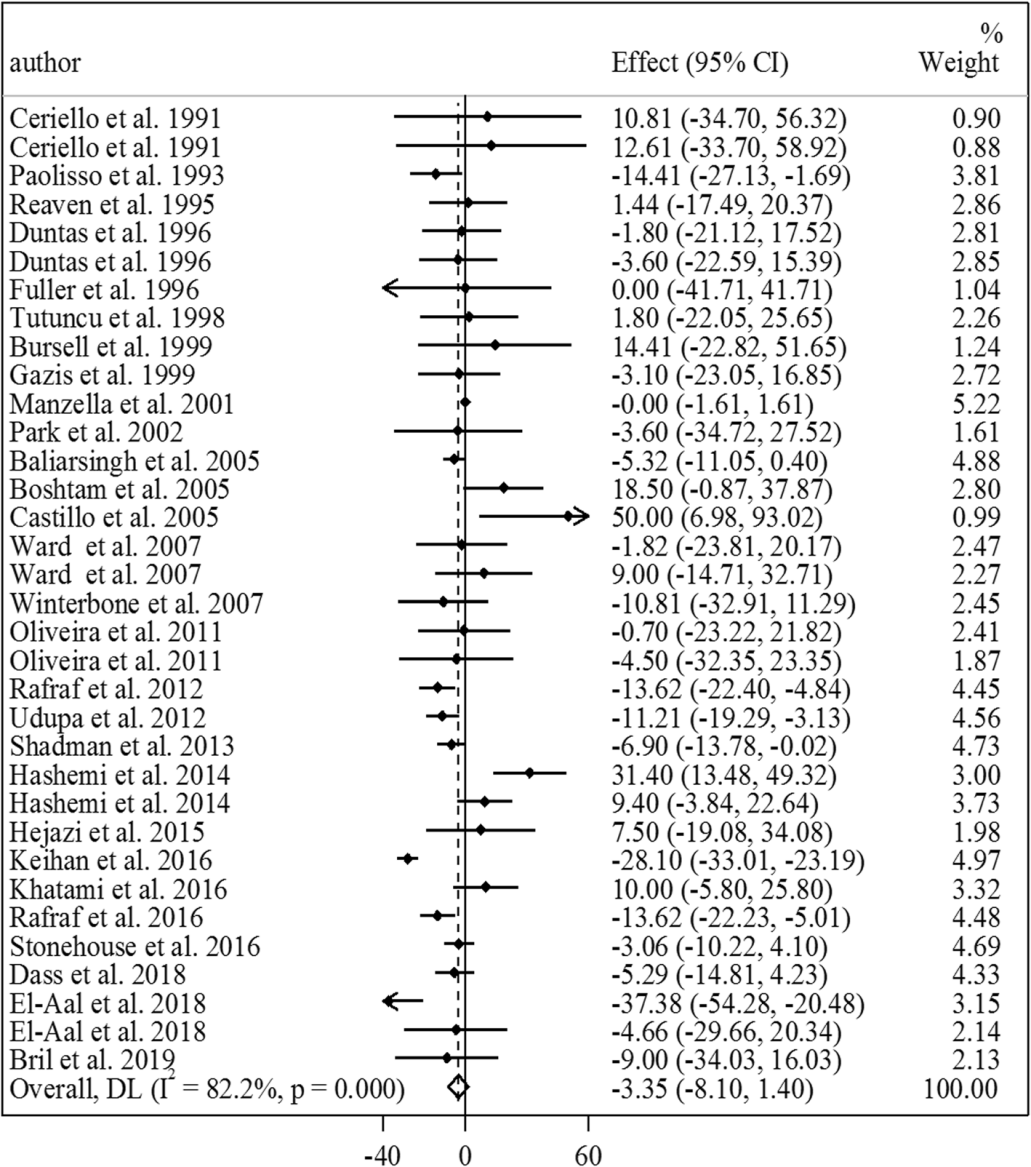
Forest plot for the effect of vitamin E intake on fasting blood glucose in diabetic patients, expressed as mean differences between intervention and control groups. Horizontal lines represent 95% CIs. Diamonds represent pooled estimates from random-effects analysis. Effect column contains weighted mean differences. CI: confidence interval

**Fig. 2:**
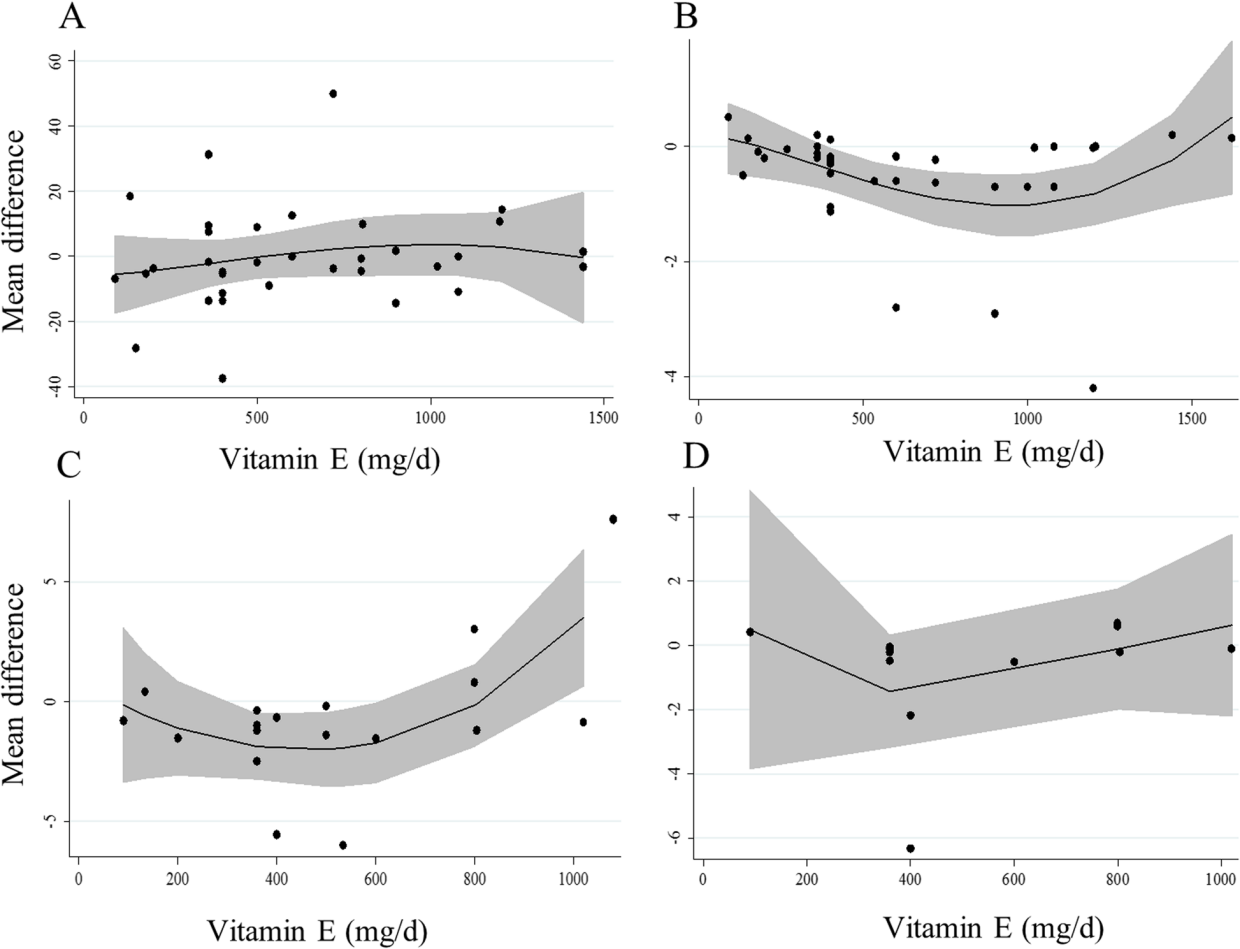
Non-linear dose–response effects of vitamin E dosages (mg/d) on (**A**) fasting blood glucose (**B**) HbA1c, (**C**) fasting insulin, and (**D**) HOMA-IR in diabetic patients. The 95% CI is demonstrated in the shaded regions. HOMA-IR: homeostatic model assessment for insulin resistance, CI: confidence interval

Overall, 28 studies with a total sample size of 1410 participants (727 patients in the vitamin E group and 683 ones in the control group) presented data on the effect of vitamin E on fasting blood glucose [7, 10–13, 17–21, 23, 25–28, 44, 45, 47–49, 51, 53–57, 60, 61]. Combining mean differences from these studies showed no significant effect of vitamin E on fasting blood glucose in diabetic patients with significant between-study heterogeneity (MD: -3.35 mg/dL, 95% CI: -8.10 to 1.40, *P* = 0.16, I^2^: 82.2%, *P* < 0.001) (Fig. 1). Subgroup analyses indicated that the heterogeneity was due to study locations, different study designs, intervention duration, types of vitamin E, vitamin E dosages, and types of diabetes (Table 2). In addition, we found that vitamin E intake resulted in a significant reduction in fasting blood glucose in studies with an intervention duration of < 10 weeks. Based on the dose–response analysis, we observed no non-linear association between vitamin E dosages and mean differences in fasting blood glucose of diabetic patients (Fig. 2. A).

**Table 2:** Subgroup analyses for the effect of vitamin E intake on glycemic indices and insulin resistance in patients with diabetes

	Effect size, *n*	WMD (95% CI)^a^	P-within^2^	*I*^b^ (%)^c^	P-heterogeneity^d^
Vitamin E intake on fasting blood glucose					
Overall	34	-3.35 (-8.10, 1.40)	0.16	82.2	< 0.001
Intervention duration (week)					
< 10	15	-6.04 (-9.88, -2.21)	0.002	19.1	0.24
≥ 10	19	-2.86 (-10.39, 4.66)	0.45	89	< 0.001
Type of vitamin E					
α-tocopherol	21	-3.75 (-11.21, 3.71)	0.32	88.4	< 0.001
Tocoterienol	2	-4.44 (-8.91, 0.03)	0.05	0.0	0.62
Mixed-tocopherols	1	9.00 (-14.72, 32.71)	0.45	0.0	-
Unclear	10	-5.24 (-9.59, -0.88)	0.01	3.3	0.40
Dosage of vitamin E (mg/day)					
< 500	15	-7.13 (-14.99, 0.73)	0.07	87.3	< 0.001
≥ 500	19	-0.22 (-1.73, 1.29)	0.77	0.0	0.55
Study location					
Western countries	16	-0.33 (-1.85, 1.19)	0.67	0.0	0.52
Non-Western countries	18	-4.53 (-11.59, 2.53)	0.20	85.1	< 0.001
Study design					
Crossover	3	-7.07 (-15.46, 1.32)	0.09	30.1	0.23
Parallel	31	-2.82 (-8.08, 2.44)	0.29	83.4	< 0.001
Blinded	25	-3.96 (-9.39, 1.47)	0.15	86.5	< 0.001
Non-blinded	9	-2.81 (-9.62, 3.99)	0.41	0	0.49
Type of diabetes					
T2DM	27	-4.42 (-9.63, 0.76)	0.09	85	< 0.001
T1DM	6	0.87 (-10.53, 12.27)	0.88	0.0	0.94
Diabetic nephropathy	1	10.00 (-5.80, 25.80)	0.21	0.0	
Risk of bias ^e^					
High	28	-4.10 (-9.81, 1.61)	0.16	83.2	< 0.001
Low	6	0.04 (-9.71, 9.78)	0.994	79.7	< 0.001
Vitamin E intake on HbA1c					
Overall	36	-0.21 (-0.33, -0.09)	0.001	76.6	< 0.001
Intervention duration (week)					
< 10	13	-0.24 (-0.48, 0.00)	0.05	75.3	< 0.001
≥ 10	23	-0.24 (-0.48, 0.00)	0.01	78.2	< 0.001
Type of vitamin E					
α-tocopherol	19	-0.18 (-0.36, 0.01)	0.06	76.3	< 0.001
Tocoterienol	6	-0.09 (-0.15, -0.04)	< 0.001	0.0	0.81
unclear	11	-0.71 (-1.21, -0.21)	0.005	86	< 0.001
Dosage of Vitamin E (mg/day)					
≥ 500	18	-0.41 (-0.68, -0.14)	0.003	80.2	< 0.001
< 500	18	-0.10 (-0.22, 0.02)	0.09	62.2	< 0.001
Study location					
Western countries	16	-0.40 (-0.69, -0.11)	0.007	79.5	< 0.001
Non-Western countries	20	-0.12 (-0.24, 0.00)	0.05	66.5	< 0.001
Study design					
Crossover	6	-0.09 (-0.15, -0.04)	0.001	0	0.54
Parallel	30	-0.26 (-0.43, -0.10)	0.002	80.1	< 0.001
Blinded	27	-0.16 (-0.29, -0.04)	0.01	74.8	< 0.001
Non-blinded	9	-0.68 (-1.20, -0.17)	0.01	82.8	< 0.001
Type of diabetes					
T2DM	23	-0.16 (-0.30, -0.02)	0.02	78.7	< 0.001
T1DM	9	-0.76 (-1.37, -0.15)	0.01	80.8	< 0.001
Diabetic nephropathy	2	-0.24 (-0.56, 0.07)	0.12	0	0.90
T2DM & T1DM	1	0.00 (-0.34, 0.34)	1.00	0	-
Diabetic neuropathy	1	-0.18 (-0.66, 0.30)	0.45	0	-
Risk of bias ^e^					
High	30	-0.28 (-0.42, -0.13)	< 0.001	79.4	< 0.001
Low	6	0.01 (-0.17, 0.18)	0.95	25.3	0.24
Vitamin E intake on fasting Insulin concentrations					
Overall	18	-1.05 (-1.53, -0.58)	< 0.001	52.7	0.005
Intervention duration (week)					
< 10	8	-0.88 (-1.27, -0.49)	< 0.001	0.0	0.80
≥ 10	10	-1.06 (-1.86, -0.26)	0.009	57.8	0.01
Type of vitamin E					
α-tocopherol	12	-0.87 (-1.60, -0.14)	0.01	53.5	0.01
Tocoterienol	1	-0.88 (-1.33, -0.43)	< 0.001	100	-
Mixed-tocopherols	1	-1.40 (-5.94, 3.14)	0.54	100	-
Unclear	4	-1.54 (-2.79, -0.29)	0.01	23.1	0.27
Dosage of Vitamin E (mg/day)					
< 500	9	-1.06 (-1.73, -0.39)	0.002	15.1	0.30
≥ 500	9	-1.00 (-1.68, -0.32)	0.004	66.1	0.003
Study location					
Western countries	6	-1.28 (-1.92, -0.64)	< 0.001	61.1	0.02
Non-Western countries	12	-0.87 (-1.56, -0.19)	0.01	35.3	0.10
Study design					
Blinded	16	-1.02 (-1.53, -0.52)	< 0.001	56.6	0.003
Non-blinded	2	-0.18 (-6.53, 6.18)	0.05	28.1	0.23
Type of diabetes					
T2DM	17	-1.03 (-1.55, -0.51)	< 0.001	55.1	0.003
Diabetic nephropathy	1	-1.20 (-2.31, -0.09)	0.03	0	-
Risk of bias ^e^					
High	12	-1.05 (-1.85, -0.24)	0.011	44.6	0.047
Low	6	-0.89 (-1.27, -0.51)	< 0.001	0	0.46
Vitamin E intake on HOMA-IR					
Overall	12	-0.44 (-0.82, -0.05)	0.02	83.4	< 0.001
Intervention duration (week)					
< 10	4	-0.18 (-0.43, 0.07)	0.15	6.5	0.36
≥ 10	8	-0.73 (-1.36, -0.08)	0.02	87.6	< 0.001
Type of vitamin E					
α-tocopherol	8	-0.72 (-1.29, -0.15)	0.01	87.5	< 0.001
Tocoterienol	1	-0.10 (-0.39, 0.19)	0.49	0	-
Unclear	3	0.0 (-0.57, 0.58)	0.98	0	0.67
Dosage of Vitamin E (mg/day)					
< 500	7	-0.98 (-1.83, -0.13)	0.02	88.7	< 0.001
≥ 500	5	-0.17 (-0.55, 0.20)	0.36	69.5	0.01
Study location					
Western countries	2	-0.33 (-0.74, 0.08)	0.11	86.0	0.007
Non-Western countries	10	-0.59 (-1.26, 0.08)	0.08	84.6	< 0.001
Type of diabetes					
T2DM	11	-0.47 (-0.88, -0.05)	0.02	84.8	< 0.001
Diabetic nephropathy	1	-0.20 (-1.03, 0.63)	0.63	0	-
Risk of bias ^e^					
High	7	-0.95 (-1.93, 0.02)	0.054	88.8	< 0.001
Low	5	-0.18 (-0.38, 0.02)	0.084	0	0.518

Abbreviations: *HbA1C* Hemoglobin A1c, *WMD* weighted mean difference, *HOMA-IR* homeostatic model assessment for insulin resistance

^a^Obtained from the random-effects model

^b^Refers to the mean (95% CI)

^c^Inconsistency, percentage of variation across studies due to heterogeneity

^d^Obtained from the Q-test

^e^If a trial had “low risk” for all domains of the Cochrane Risk of Bias Assessment tool, it was considered a high-quality study with a totally low risk of bias

Sensitivity analysis revealed that the overall non-significant effect of vitamin E on fasting blood glucose did not depend on any studies. Also, excluding the RCTs with a combination treatment from the overall analysis led to no changes in the non-significant effect of vitamin E on fasting blood glucose (MD: -2.32 mg/dL, 95% CI: -7.55 to 2.91, *P* = 0.38, I^2^: 83.0%, *P* < 0.001) (Supplemental Fig. 2). Regarding publication bias, the Egger regression test rejected our hypothesis about the presence of substantial publication bias (*P* = 0.76).

**Fig. 3:**
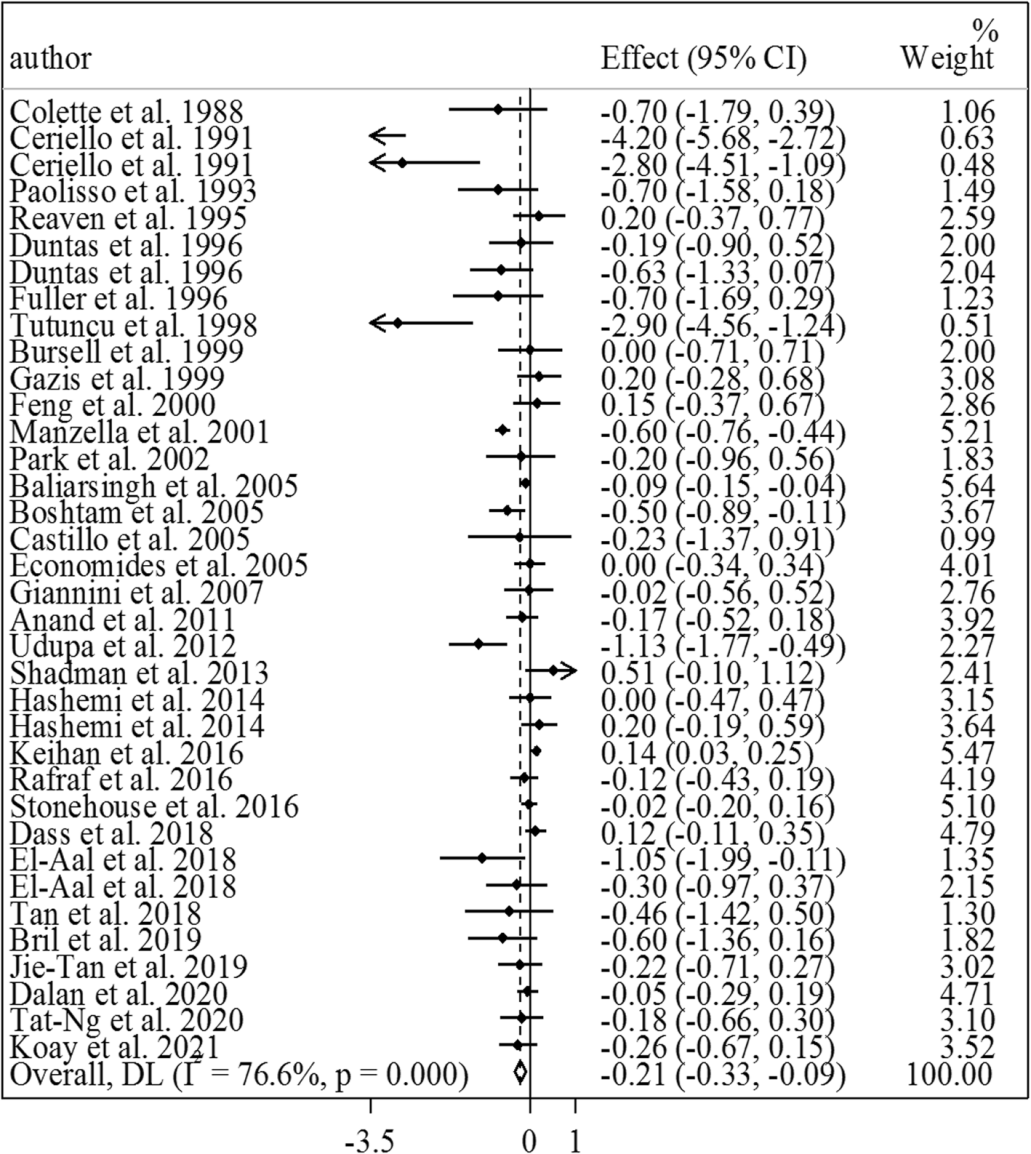
Forest plot for the effect of vitamin E intake on HbA1c in diabetic patients, expressed as mean differences between intervention and control groups. Horizontal lines represent 95% CIs. Diamonds represent pooled estimates from random-effects analysis. Effect column contains weighted mean differences. CI: confidence interval

Findings from the meta-analysis on vitamin E and HbA1c: Of included RCTs, 32 studies that enrolled 1737 participants (883 in the vitamin E group and 854 in the control group) were included in this Sect. [7, 10, 12–16, 18, 20, 21, 23–26, 28, 29, 44, 46, 48–61]. Meta-analysis of these RCTs showed that vitamin E intake, compared with a placebo, resulted in a significant reduction in HbA1c in diabetic patients (MD: -0.21%, 95% CI: -0.33 to -0.09, *P* = 0.001, I^2^: 76.6%, *P* < 0.001) (Fig. 3). However, we found significant heterogeneity among the studies. Based on the subgroup analyses, this heterogeneity was due to study designs, types of vitamin E prescribed, types of diabetes, and risk of bias among included studies (Table 2). From these analyses, a significant reducing effect of vitamin E intake on HbA1c was found in all subgroups of studies, except for those studies that recruited patients with diabetic nephropathy, those that used vitamin E in the form of alpha-tocopherol for the intervention, RCTs prescribing < 500 mg/day vitamin E, and those studies with a high risk of bias. Of note, in these subgroups, the estimates were marginally significant (Table 2). In the non-linear dose–response analysis, we found that the most efficient range for vitamin E dosages to lower HbA1c in diabetic patients was between 400 and 1300 mg/day, while the highest level of efficiency was reported with the dosage of 1000 mg/day (Fig. 2.B).

According to the sensitivity analysis, we found that the overall estimate obtained for the effect of vitamin E on HbA1c did not depend on a single study. In addition, after excluding the RCTs with combined treatment, the lowering effect of vitamin E on HbA1c remained significant (MD: -0.23%, 95% CI: -0.36 to -0.10, *P* = 0.001, I^2^: 77.8%, *P* < 0.001) (Supplemental Fig. 3). Based on Egger’s regression test, we found significant publication bias for the overall effect (*P* = 0.02). However, by filling the possibly missed studies using the trim-and-fill method, the significance of the effect of vitamin E to lower HbA1c did not change.

**Fig. 4:**
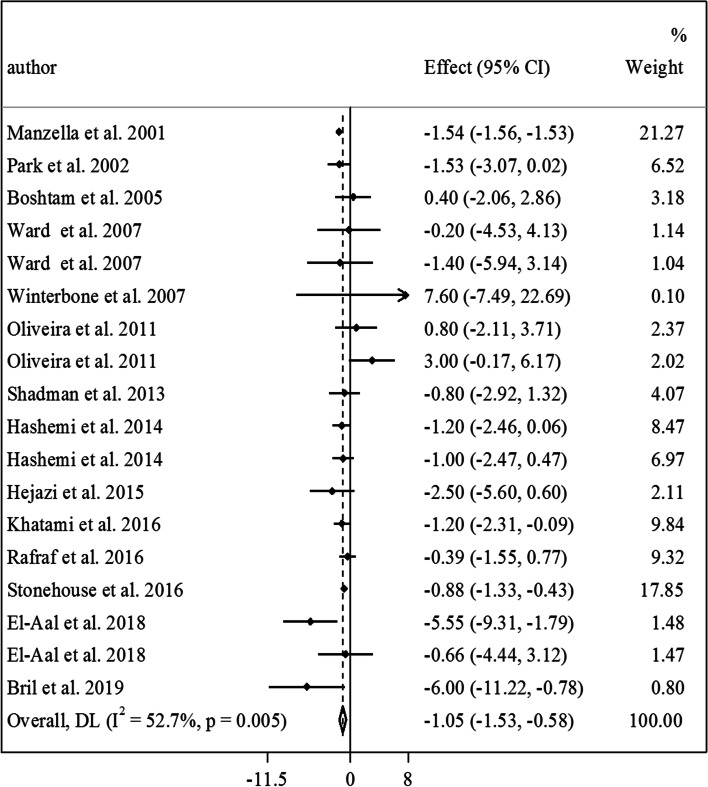
Forest plot for the effect of vitamin E intake on fasting insulin in diabetic patients, expressed as mean differences between intervention and control groups. Horizontal lines represent 95% CIs. Diamonds represent pooled estimates from random-effects analysis. Effect column contains weighted mean differences. CI: confidence interval

Findings from the meta-analysis on vitamin E and fasting insulin: In total, 13 RCTs with a total sample size of 791 subjects (401 in the vitamin E group and 390 in the control group) were included for vitamin E and fasting insulin [12, 17–19, 21, 23, 26–28, 45, 47, 49, 51, 60]. After combining the results from these studies, we found a significant lowering effect of vitamin E intake, compared with a placebo, on fasting insulin in diabetic patients (MD: -1.05 µIU/mL, 95% CI: -1.53 to -0.58, *P* < 0.001, I^2^: 52.7%, *P* = 0.005) (Fig. 4). Between-study heterogeneity was significant in this analysis. Subgroup analyses based on study locations, intervention duration, types of vitamin E, and studies’ risk of bias could explain the observed heterogeneity (Table 2). Also, the significant reducing effect of vitamin E was seen in all subgroups of studies, except in those that did not do blinding in their interventions. Moreover, in the dose–response analysis, we found a reducing effect of vitamin E on fasting insulin in the dosages between 400 and 700 mg/day of vitamin E. However, out of this range, no significant effect was observed (Fig. 2.C).

In the sensitivity analysis, excluding any studies from the meta-analysis did not change our findings on the effect of vitamin E intake on the fasting level of insulin. Such finding was also seen after excluding the RCTs with a combination treatment (MD: -1.12 µIU/mL, 95% CI: -1.57 to -0.67, *P* < 0.001, I^2^: 46.7%, *P* = 0.03) (Supplemental Fig. 4). Although Egger’s regression test showed marginally significant publication bias in the meta-analysis (*P* = 0.06), filling the possibly missed studies using the trim-and-fill method resulted in no changes in the significant effect of vitamin E in lowering the fasting level of insulin.

**Fig. 5:**
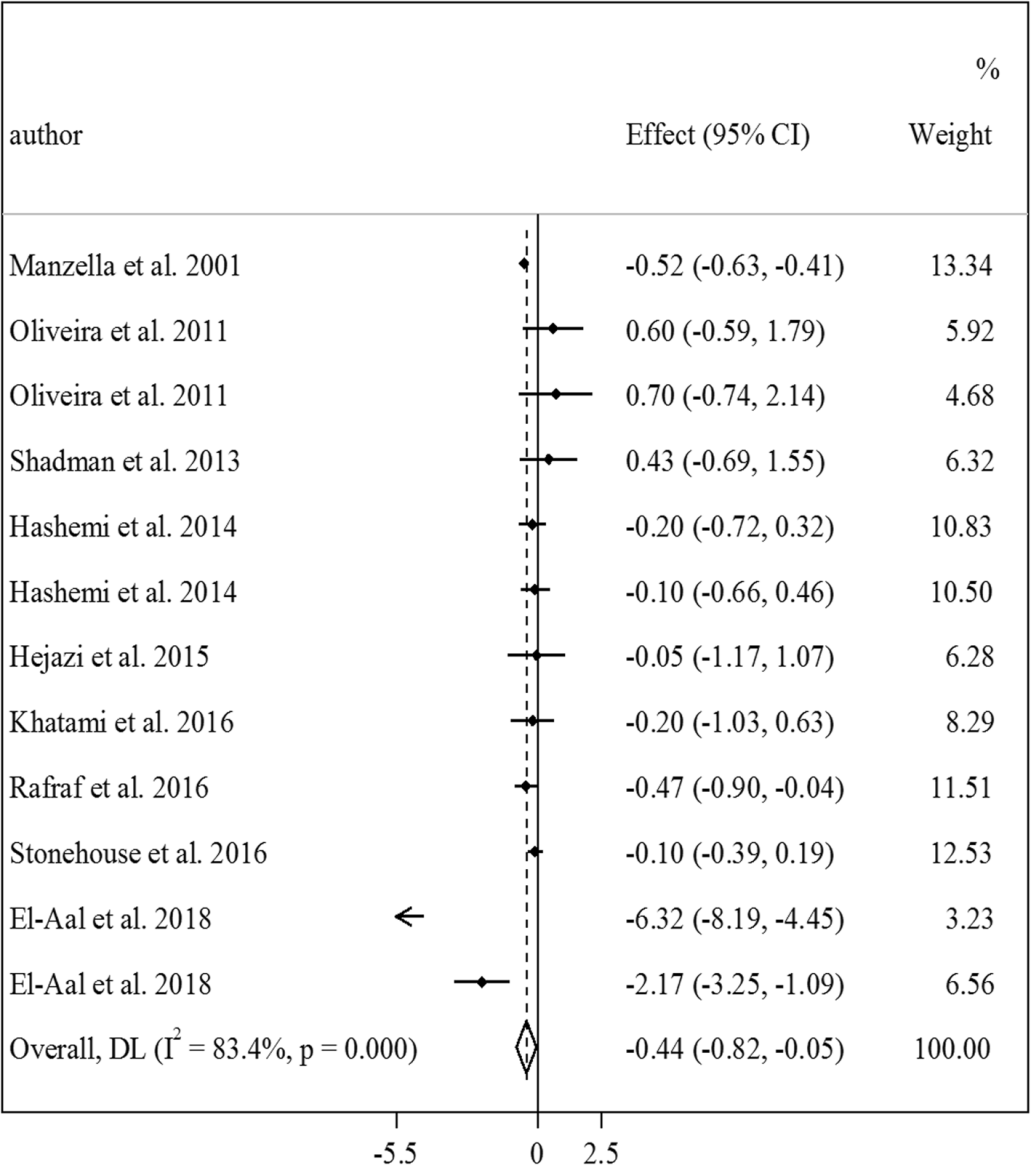
Forest plot for the effect of vitamin E intake on HOMA-IR in diabetic patients, expressed as mean differences between intervention and control groups. Horizontal lines represent 95% CIs. Diamonds represent pooled estimates from random-effects analysis. Effect column contains weighted mean differences. HOMA-IR: homeostatic model assessment for insulin resistance, CI: confidence interval

Findings from the meta-analysis on vitamin E and HOMA-IR: Nine studies with a total sample size of 462 diabetic patients (223 patients in the vitamin E group and 239 patients in the control group) were included in the meta-analysis of vitamin E and HOMA-IR [12, 17, 18, 21, 26–28, 45, 51]. Combining results of these studies revealed that vitamin E intake reduced HOMA-IR in diabetic patients (MD: -0.44, 95% CI: -0.82 to -0.05, *P* = 0.02, I^2^: 83.4%, *P* < 0.001) (Fig. 5). However, between-study heterogeneity was high in this meta-analysis. Based on the subgroup analyses, the heterogeneity could be due to the intervention duration, types of vitamin E prescribed, and studies’ risk of bias (Table 2). Moreover, a significant lowering effect was seen in studies that used alpha-tocopherol as the intervention, those with an intervention duration of ≥ 10 weeks, RCTs that prescribed < 500 mg/day of vitamin E, and studies that included patients with type 2 diabetes mellitus. In the dose–response analysis, no significant association was seen between vitamin E dosage and changes in HOMA-IR in diabetic patients (Fig. 2.D).

In the sensitivity analysis, we found no dependency of overall estimate on a single study. Moreover, excluding the RCTs with a combination treatment did not lead to any changes in the significant effect of vitamin E on HOMA-IR (MD: -0.30, 95% CI: -0.53 to -0.06, *P* = 0.01, I^2^: 50.3%, *P* = 0.06) (Supplemental Fig. 5). In terms of publication bias, the Egger regression test did not show substantial publication bias (*P* = 0.97).

## Discussion

In the current study, we found that vitamin E intake resulted in a significant reduction in HbA1c, fasting insulin, and HOMA-IR in diabetic patients. In terms of fasting blood glucose, we found no significant effect in the overall analysis; however, in the subgroup analyses, a significant lowering effect was seen for vitamin E in studies with an intervention duration of < 10 weeks.

Different approaches such as pharmacological methods, diet modification, and using a high dose of nutritional supplements have been proposed for controlling hyperglycemia in patients with diabetes [3]. Although recent RCTs have shown that intake of vitamin E supplements affects glycemic indices and insulin resistance in patients with hyperglycemia, findings from these RCTs are conflicting. In the current meta-analysis of RCTs, we concluded that vitamin E intake significantly reduced HbA1c in diabetic patients. However, in another meta-analysis, which was published in 2014, Xu et al. reported a different result [22]. They concluded that vitamin E intake had no significant effect on HbA1c. In the meta-analysis of Xu et al., results from diabetic patients were combined with those obtained from the general population which could explain the observed controversy. Another reason is that Xu et al. did not include nine relevant RCTs, published before the release of their meta-analysis, that most of them reported a beneficial effect of vitamin E on glycemic indices [11, 13, 46, 47, 52, 53, 55, 56, 59].

In the present study, we found no significant effect of vitamin E on fasting blood glucose in the overall analysis which was in contrast with our findings on HbA1c. Prior evidence suggested that vitamin E interferes with protein glycosylation in the Maillard reaction [62]. And, as glucose oxidation is a necessary step for hemoglobin modification by glucose [7], its’ inhibition will in turn be resulted in the reduction of the covalent linking between glucose, albumin and hemoglobin and thus reduces the total protein glycosylation [63]. It is proposed that vitamin E may reduce HbA1c through the inhibition of glucose oxidation [28]. In addition, it seems that blood glucose is susceptible to short-term interventions, while HbA1c is mostly affected by long-term interventions [44]. This is in line with our findings from the subgroup analyses that vitamin E intake only reduced fasting blood glucose in studies with an intervention duration of < 10 weeks and HbA1c in studies with an intervention duration of ≥ 10 weeks.

In the present meta-analysis, vitamin E intake had a reducing effect on fasting insulin and HOMA-IR. Both insulin levels and HOMA-IR are indicators of insulin resistance. In a narrative review, Tosatti et al. concluded that adherence to a Mediterranean diet, known as a vitamin-E-rich diet, beneficially affects insulin resistance and glucose metabolism in patients with T2DM [64]. In contrast, the meta-analysis of Xu et al. showed no significant effect of vitamin E on fasting insulin in the general population [22]. Different health conditions of the study population and different quality of previous studies might be among the reasons for this contradiction. For instance, in the subgroup analyses, we found a significant lowering effect of vitamin E intake on fasting insulin in the studies in which participants were blinded to the intervention, while this effect was not seen in non-blinded RCTs. Previous studies have also demonstrated a possible mechanistic link between vitamin E and insulin sensitivity. They reported that alpha- and gamma-tocopherol upregulate an endogenous ligand involved in activating PPARγ which plays an important role in the upregulation of adiponectin. This endogenous adipokine has also been shown to enhance insulin sensitivity [65].

In the dose–response analysis, we found that the most efficient range of vitamin E dosage for reducing HbA1c in diabetic patients is between 500 and 1300 mg/day and for reducing fasting insulin is between 400 and 700 mg/day. Considering both effects, it seems that the best dosages are between 400 to 700 mg/day and at these dosages, no adverse effect of vitamin E on glycemic indices was reported. It should be noted that these doses must be consumed along with regular dietary intake of vitamin E. However, intake of a high dose of vitamin E (> 1300 mg/day) not only has a significant effect but may also have an adverse effect on glycemic indices. It might be explained by the paradoxical effects of vitamin E in different dosages. There is evidence that a relatively high dose of vitamin E cannot inhibit the process of oxidative stress which is involved in hemoglobin glycosylation [66]. Overall, it seems that a low dose of vitamin E for supplementation is more effective than a high dose in diabetic patients. However, further studies are needed to clarify this issue.

Out of 38 studies included in the current meta-analysis, only 5 studies had a low risk of bias and most of the studies had a high risk of bias in “selective reporting” and “Other sources of bias (considering dietary intake of vitamin E during the trial)” items since they assessed only one of the glycemic indices in blood and did not control dietary intakes of vitamin E throughout the trial. In the subgroup analyses based on overall risk of bias (high vs. low), our findings regarding all glycemic indices, except for HbA1c, did not change between the two subgroups. For HbA1c, we found a significant lowering effect of vitamin E supplementation in the overall analysis and sub-group analysis of studies with a high risk of bias, while such a significant effect was not observed among studies with a low risk of bias. Therefore, our findings on the beneficial effect of vitamin E on HbA1c should be cautiously considered. Further studies are also required to assess this effect.

It must be kept in mind that the effects of vitamin E might be changed when it was administered with other therapeutic strategies used for diabetic patients. For instance, Afzali et al. reported that magnesium and vitamin E co-supplementation for 12 weeks had a reducing effect on fasting blood glucose, triglycerides, LDL- and HDL-cholesterol, hs-CRP, and oxidative stress [67]. Also, in a meta-analysis, Li et al. reported that omega-3 fatty acid and vitamin co-supplementation may have a favorable effect on metabolic status in gestational diabetes [68]. However, in the current meta-analysis, vitamin E intake alone had no significant effect on blood glucose. Therefore, the effects of vitamin E in combination with other nutrients or drugs should be assessed in future RCTs.

The current meta-analysis had some strengths. This meta-analysis was the first study that summarized available findings on the effect of vitamin E intake on glycemic indices and insulin resistance in diabetic patients by considering all available RCTs. Also, we assessed the dose–response association between vitamin E dosages and changes in glycemic indices. In addition, we performed the meta-analysis by using a random-effects model which takes between-study heterogeneity into account. Some limitations in this meta-analysis and among included RCTs should be also considered when interpreting the present results. In the current meta-analysis, the heterogeneity was high in the overall analyses; however, we tried to control it by performing the analyses using a random-effects model. Also, we found potential sources of heterogeneity in the subgroup analyses. In some analyses, we found publication bias; however, excluding this bias using the application of trim-and-fill did not change our findings. Regarding the limitations of included RCTs, they used different types of tocopherols for the intervention which might affect our findings. Future studies are recommended to focus on the most effective types of tocopherols. Moreover, different study designs and not controlling for baseline measures in some other studies must also be counted as further limitations. In addition, the majority of RCTs had a high risk of bias that can affect the reliability of our findings.

## Conclusion

We found that vitamin E intake significantly reduces levels of HbA1c, fasting insulin, and HOMA-IR in diabetic patients, particularly patients with T2DM. Also, a significant reducing effect of vitamin E intake on fasting blood glucose was found in studies with an intervention duration of < 10 weeks. Moreover, we found that the best vitamin E dosages for controlling HbA1c and insulin levels are between 400 and 700 mg/day. Overall, since no known side effects were reported for vitamin E supplementation, its intake with dosages of 400 to 700 mg/day is recommended for patients with T2DM. Therefore, vitamin E can be presented as a supplementary treatment along with the main treatments (i.e. medications) for these patients. However, this recommendation should be done with caution for patients with T1DM and those with diabetic nephropathy and/or neuropathy, as only a few studies are available in this regard. Further RCTs, particularly those with a low risk of bias, are needed to assess the effect of vitamin E supplementation on biochemical parameters of patients with T1DM and those with diabetic nephropathy and/or neuropathy.

## Supplementary Information

**Additional file 1: Supplemental Table 1. **The terms used tosearch relevant publications on the effect of vitamin E intake on glycemicindices and insulin resistance in patients with diabetes. **Supplemental Table 2.** Results of risk of bias assessment for randomized clinical trials included in the currentmeta-analysis on the effectsof vitamin E supplementation on glycemic indices and insulin resistance inpatients with diabetes mellitus^1^. **Supplemental Figure 1. **Flow diagram of studyselection. **Supplemental Figure 2.** Forest plot for theeffect of vitamin E intake on fasting blood glucose in diabetic patients afterexcluding RCTs with a combination treatment. Effect column expresses meandifferences between intervention and control groups. Horizontal lines represent95% CIs. Diamonds represent pooled estimates from random-effects analysis. CI:confidence interval, RCTs: randomized controlled trials. **Supplemental Figure 3.** Forest plot for theeffect of vitamin E intake on HbA1c in diabetic patients after excluding RCTswith a combination treatment. Effect column expresses mean differences betweenintervention and control groups. Horizontal lines represent 95% CIs. Diamondsrepresent pooled estimates from random-effects analysis. CI: confidenceinterval, RCTs: randomized controlled trials. **Supplemental Figure 4.** Forest plot for the effect of vitamin E intake on fasting insulin in diabetic patients afterexcluding RCTs with a combination treatment. Effect column expresses meandifferences between intervention and control groups. Horizontal lines represent95% CIs. Diamonds represent pooled estimates from random-effects analysis. CI:confidence interval, RCTs: randomized controlled trials. **Supplemental Figure 5.** Forest plot for theeffect of vitamin E intake on HOMA-IR in diabetic patients after excluding RCTs with a combination treatment. Effect column expresses mean differences betweenintervention and control groups. Horizontal lines represent 95% CIs. Diamondsrepresent pooled estimates from random-effects analysis. CI: confidenceinterval, RCTs: randomized controlled trials.

## Data Availability

The datasets analyzed during the current study are presented in the manuscript.
