# Supplementary material for: Effect of vitamin E intake on glycemic control and insulin resistance in diabetic patients: an updated systematic review and meta-analysis of randomized controlled trials
Source: Nutr J. 2023 Feb 17;22:10. doi: 10.1186/s12937-023-00840-1 (PMC9936725; doi:10.1186/s12937-023-00840-1)
Supplement: Supplementary file 1 — Additional file 1: Supplemental Table 1. The terms used tosearch relevant publications on the effect of vitamin E intake on glycemicindices and insulin resistance in patients with diabetes. Supplemental Table 2. Results of risk of bias assessment for randomized clinical trials included in the currentmeta-analysis on the effectsof vitamin E supplementation on glycemic indices and insulin resistance inpatients with diabetes mellitus1. Supplemental Figure 1. Flow diagram of studyselection. Supplemental Figure 2. Forest plot for theeffect of vitamin E intake on fasting blood glucose in diabetic patients afterexcluding RCTs with a combination treatment. Effect column expresses meandifferences between intervention and control groups. Horizontal lines represent95% CIs. Diamonds represent pooled estimates from random-effects analysis. CI:confidence interval, RCTs: randomized controlled trials. Supplemental Figure 3. Forest plot for theeffect of vitamin E intake on HbA1c in diabetic patients after excluding RCTswith a combination treatment. Effect column expresses mean differences betweenintervention and control groups. Horizontal lines represent 95% CIs. Diamondsrepresent pooled estimates from random-effects analysis. CI: confidenceinterval, RCTs: randomized controlled trials. Supplemental Figure 4. Forest plot for the effect of vitamin E intake on fasting insulin in diabetic patients afterexcluding RCTs with a combination treatment. Effect column expresses meandifferences between intervention and control groups. Horizontal lines represent95% CIs. Diamonds represent pooled estimates from random-effects analysis. CI:confidence interval, RCTs: randomized controlled trials. Supplemental Figure 5. Forest plot for theeffect of vitamin E intake on HOMA-IR in diabetic patients after excluding RCTs with a combination treatment. Effect column expresses mean differences betweenintervention and control groups. Horizontal lines represent 95% CIs. Diamondsrepresent pooled estimates from [file 12937_2023_840_MOESM1_ESM.docx]

**Online Supporting Material**

**Supplemental Table 1:** The terms used to search relevant publications on the effect of vitamin E intake on glycemic indices and insulin resistance in patients with diabetes

| Concept 1 | "Vitamin E" OR "alpha-tocopherol" OR "α-tocopherol" OR "Vit E" OR tocopherol OR tocoterienol |
| --- | --- |
| Concept 2 | "Type 2 diabete*" OR T2DM OR diabete* |
| Concept 3 | Intervention OR "Intervention Study" OR "Intervention Studies" OR "controlled trial" OR randomized OR randomized OR random OR randomly OR placebo OR "clinical trial" OR Trial OR "randomized controlled trial" OR "randomized clinical trial" OR RCT OR blinded OR "double blind" OR "double blinded" OR trial OR "clinical trial" OR trials OR "pragmatic clinical trial" OR "cross-over" OR "cross over" OR parallel |
| Search | Concept 1 AND concept 2 AND concept 3 |

**Online Supporting Material**

**Supplemental Table 2**: Results of risk of bias assessment for randomized clinical trials included in the current meta-analysis on the effects of vitamin E supplementation on glycemic indices and insulin resistance in patients with diabetes mellitus^1^

| Studies | Random Sequence Generation | Allocation concealment | Selective reporting | Other sources of bias (considering dietary vitamin E during the trial) | Blinding (participants and personnel) | Blinding (outcome assessment) | Incomplete outcome data | Overall risk |
| --- | --- | --- | --- | --- | --- | --- | --- | --- |
| Koay et al. 2021 | U | L | H | H | L | U | L | High |
| Tat-Ng et al. 2020 | L | L | H | H | L | U | L | High |
| Dalan et al. 2020 | L | L | H | L | L | U | L | High |
| Tan et al. 2019 | L | L | H | H | L | U | L | High |
| Bril et al. 2019 | L | L | L | L | L | U | L | Low |
| El-Aal et al. 2018 | L | H | L | H | L | H | L | High |
| Tan et al. 2018 | L | L | H | L | L | L | L | High |
| Dass et al. 2018 | L | L | H | H | H | H | L | High |
| Rafraf et al. 2016 | L | L | L | L | L | L | L | Low |
| Stonehouse et al. 2016 | L | U | L | H | L | L | L | Low |
| Keihan et al. 2016 | L | H | H | H | L | H | L | High |
| Khatami et al. 2016 | L | L | H | H | L | U | L | High |
| Hejazi et al. 2015 | L | L | L | H | H | H | L | High |
| Hashemi et al. 2014 | L | L | L | L | L | L | L | Low |
| Shadman et al. 2013 | L | L | L | L | L | L | L | Low |
| Rafraf et al. 2012 | L | U | H | L | L | L | L | High |
| Udupa et al. 2012 | L | L | H | H | L | L | L | High |
| Vijayakumar et al. 2011 | L | U | H | H | H | H | L | High |
| Oliveira et al. 2011 | L | U | H | H | L | L | L | High |
| Giannini et al. 2007 | L | L | H | H | L | L | L | High |
| Winterbone et al. 2007 | L | U | H | H | L | U | L | High |
| Ward et al. 2007 | L | U | H | H | L | L | L | High |
| Ward et al. 2007 | L | L | H | H | L | L | L | High |
| Baliarsingh et al. 2005 | L | H | H | L | L | U | L | High |
| Boshtam et al. 2005 | L | U | H | H | L | H | L | High |
| Ble-Castillo et al. 2005 | L | U | L | H | H | H | L | High |
| Economides et al. 2005 | L | L | U | H | H | H | L | High |
| Manzella et al. 2001 | L | U | L | H | L | L | L | High |
| Park et al. 2001 | L | L | U | H | H | H | L | High |
| Feng et al. 2000 | L | L | H | H | L | L | L | High |
| Bursell et al. 1999 | L | L | H | H | L | U | L | High |
| Gazis et al. 1999 | L | L | H | H | L | U | L | High |
| Tutuncu et al. 1998 | L | L | H | H | L | L | L | High |
| Colette et al. 1998 | L | H | H | H | L | H | L | High |
| Duntas et al. 1996 | L | U | H | L | L | U | L | High |
| Fuller et al. 1996 | L | U | H | H | L | U | L | High |
| Reaven et al. 1995 | L | H | H | H | L | H | L | High |
| Paolisso et al. 1993 | L | H | L | H | L | H | L | High |
| Ceriello et al. 1991 | L | U | H | L | L | U | L | High |

^1^Each study was assessed for risk of bias using the Cochrane Risk of Bias Assessment tool. Domains of assessment were included random sequence generation, allocation concealment, reporting bias, performance bias, detection bias, attrition bias and other sources of bias. Each domain was scored as “high risk” if it contained methodological flaws that may have affected the results, “low risk” if the flaw was deemed inconsequential, and “unclear risk” if information was insufficient to determine. If a study got “low risk” for all domains, it considered as a high quality study with totally low risk of bias.

**Online Supporting Material**

**
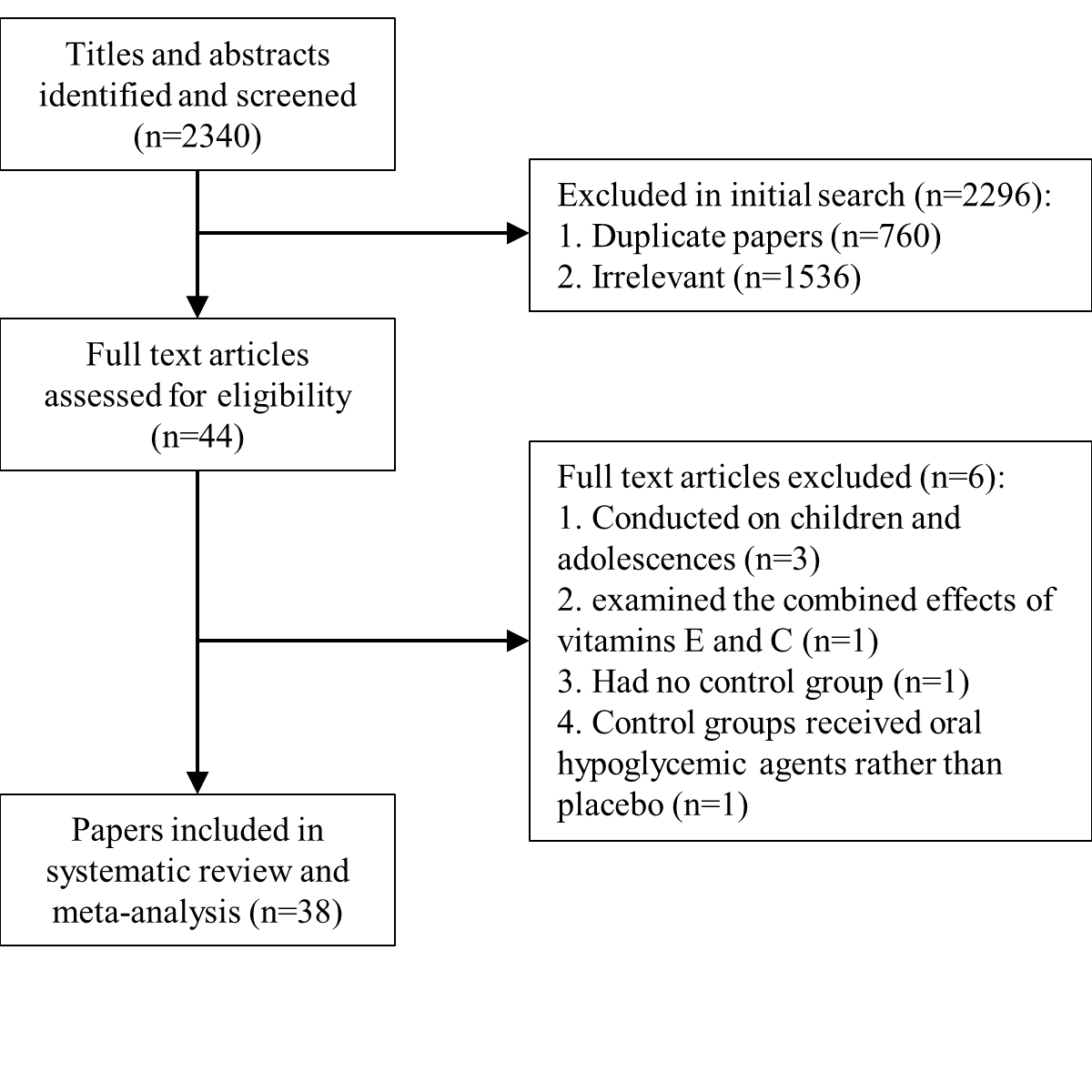
**

**Supplemental Figure 1:** Flow diagram of study selection

**Online Supporting Material**


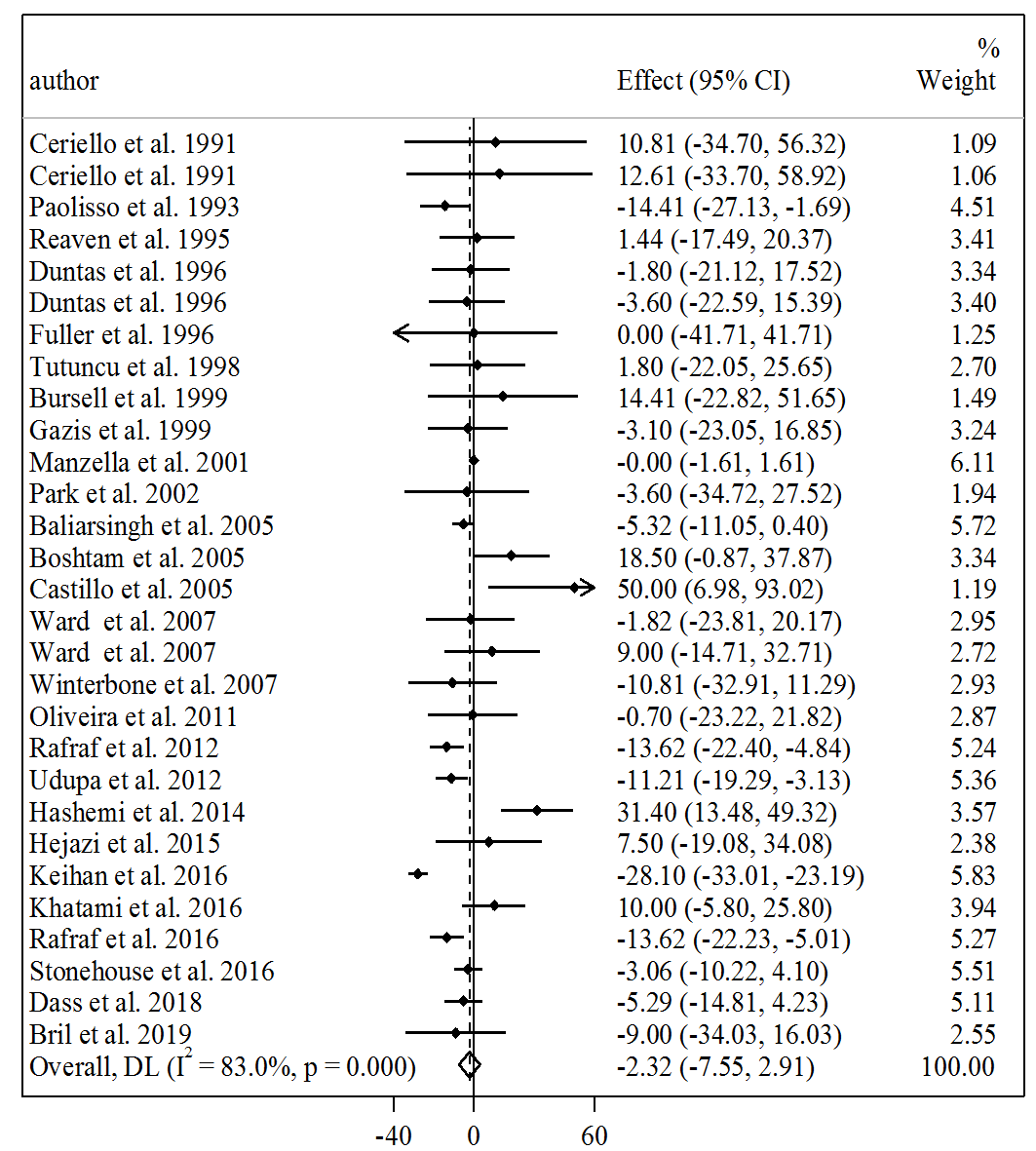


**Supplemental Figure 2**: Forest plot for the effect of vitamin E intake on fasting blood glucose in diabetic patients after excluding RCTs with a combination treatment. Effect column expresses mean differences between intervention and control groups. Horizontal lines represent 95% CIs. Diamonds represent pooled estimates from random-effects analysis. CI: confidence interval, RCTs: randomized controlled trials

**Online Supporting Material**


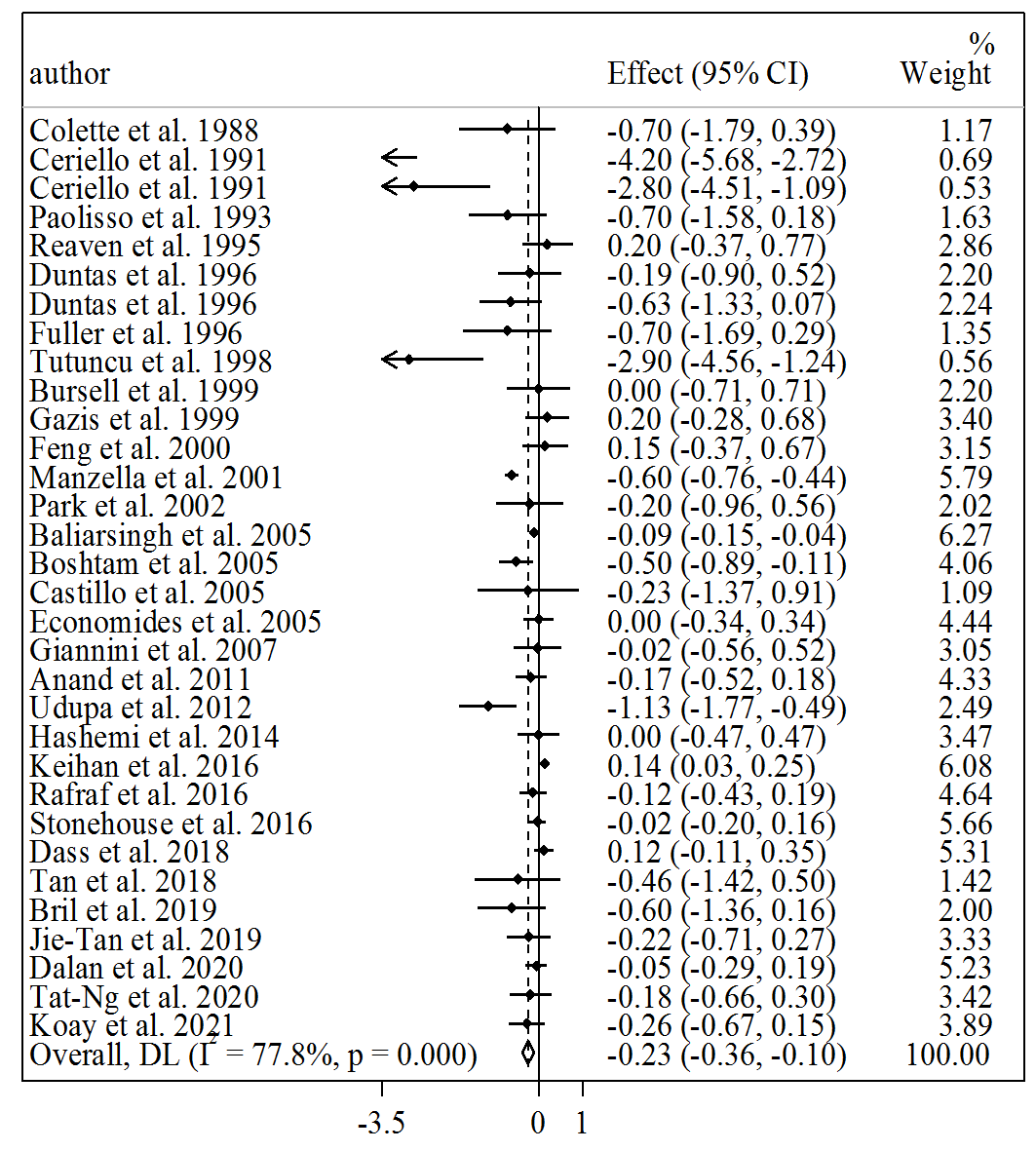


**Supplemental Figure 3**: Forest plot for the effect of vitamin E intake on HbA1c in diabetic patients after excluding RCTs with a combination treatment. Effect column expresses mean differences between intervention and control groups. Horizontal lines represent 95% CIs. Diamonds represent pooled estimates from random-effects analysis. CI: confidence interval, RCTs: randomized controlled trials

**Online Supporting Material**


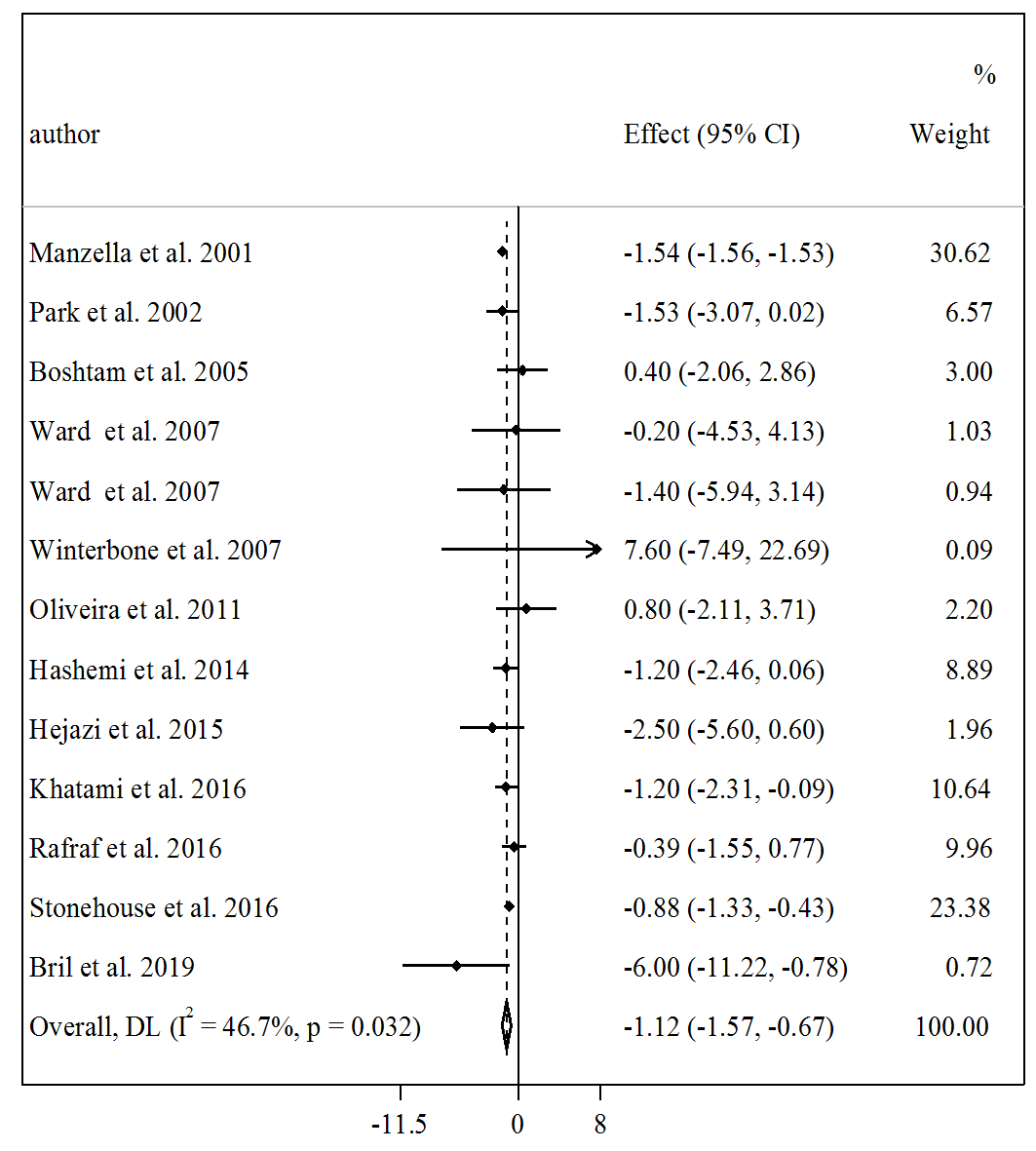


**Supplemental Figure 4**: Forest plot for the effect of vitamin E intake on fasting insulin in diabetic patients after excluding RCTs with a combination treatment. Effect column expresses mean differences between intervention and control groups. Horizontal lines represent 95% CIs. Diamonds represent pooled estimates from random-effects analysis. CI: confidence interval, RCTs: randomized controlled trials

**Online Supporting Material**


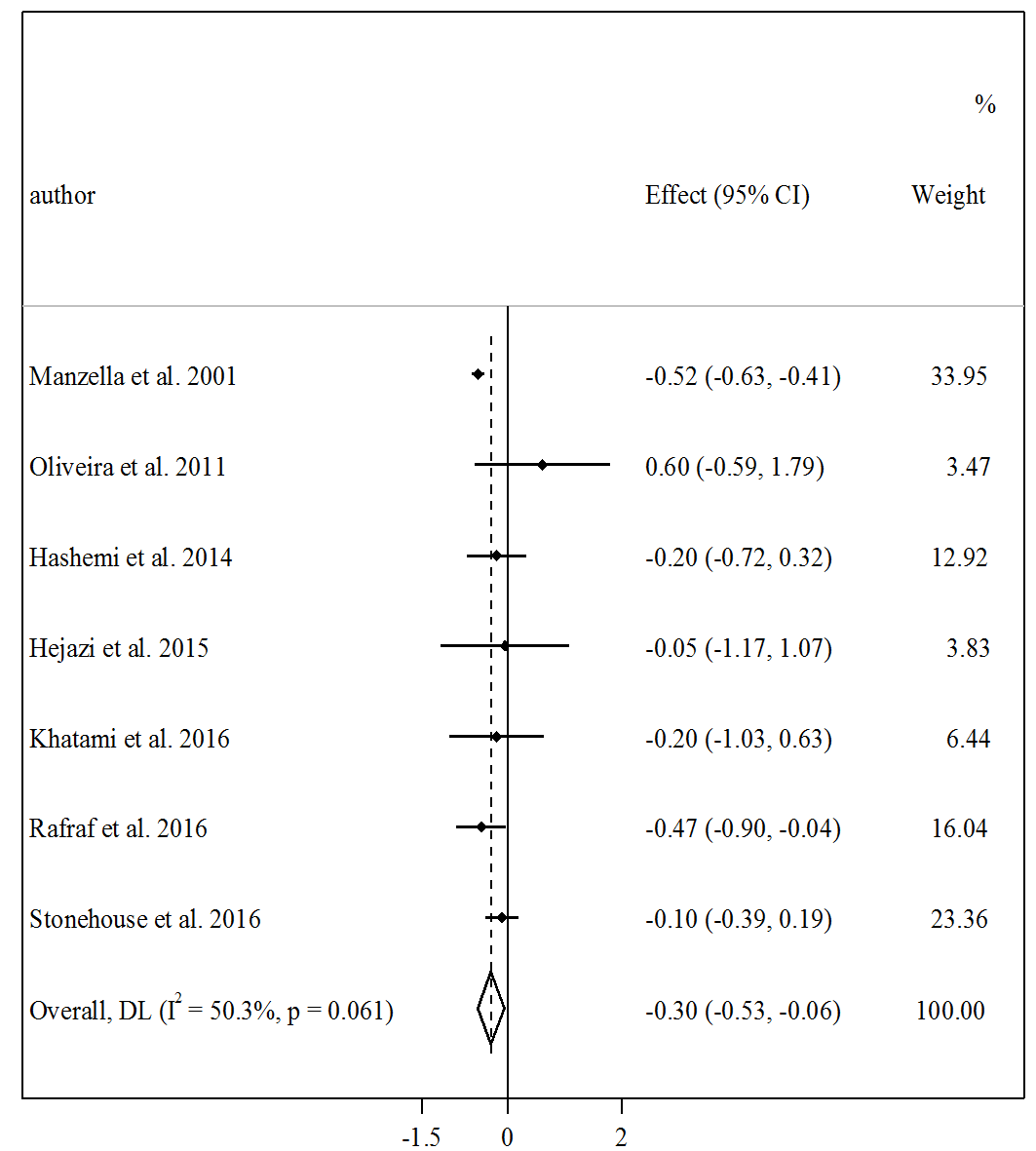


**Supplemental Figure 5**: Forest plot for the effect of vitamin E intake on HOMA-IR in diabetic patients after excluding RCTs with a combination treatment. Effect column expresses mean differences between intervention and control groups. Horizontal lines represent 95% CIs. Diamonds represent pooled estimates from random-effects analysis. CI: confidence interval, RCTs: randomized controlled trials
